# Neuronal coding of multiscale temporal features in communication sequences within the bat auditory cortex

**DOI:** 10.1038/s42003-018-0205-5

**Published:** 2018-11-20

**Authors:** Francisco García-Rosales, M. Jerome Beetz, Yuranny Cabral-Calderin, Manfred Kössl, Julio C. Hechavarria

**Affiliations:** 1 Institut für Zellbiologie und Neurowissenschaft, Goethe-Universität, 60438 Frankfurt/M., Germany; 2 MEG Labor, Brain Imaging Center, Goethe-Universität, 60528 Frankfurt/M., Germany; 3 German Resilience Center, University Medical Center Mainz, 55131 Mainz, Germany; 4 Present Address: Department of Zoology II, University of Würzburg, Am Hubland, 97074 Würzburg, Germany

## Abstract

Experimental evidence supports that cortical oscillations represent multiscale temporal modulations existent in natural stimuli, yet little is known about the processing of these multiple timescales at a neuronal level. Here, using extracellular recordings from the auditory cortex (AC) of awake bats (*Carollia perspicillata*), we show the existence of three neuronal types which represent different levels of the temporal structure of conspecific vocalizations, and therefore constitute direct evidence of multiscale temporal processing of naturalistic stimuli by neurons in the AC. These neuronal subpopulations synchronize differently to local-field potentials, particularly in theta- and high frequency bands, and are informative to a different degree in terms of their spike rate. Interestingly, we also observed that both low and high frequency cortical oscillations can be highly informative about the listened calls. Our results suggest that multiscale neuronal processing allows for the precise and non-redundant representation of natural vocalizations in the AC.

## Abstract (web summary)

García-Rosales et al. identified three distinct neuronal populations within the auditory cortex of awake *Carollia perspicillata* bats. These neurons responded to different temporal features of communication calls from conspecifics and synchronized to distinct cortical oscillations, suggesting multiscale temporal representation at a cellular level.

## Introduction

Acoustic communication is highly important in the animal kingdom since it provides a tool for transmitting relevant information about the inner state of the broadcaster and/or about its immediate environment^1–6^. Similar to human speech^7,8^, numerous animal species (e.g. birds, bats, and non-human primates) emit communication calls with multiscale temporal structures. These calls, often referred to as sequences, are typically composed of individual acoustic elements like syllables, or groups of syllables, which ultimately make up whole vocalizations^4,9–11^. For example, in bats, syllabic structures have been observed in the context of distress calling^2,12,13^. Syllables in distress vocalizations are typically repeated at short intervals of ~14 ms, and can be combined to form multi-syllabic bouts repeated at ~80 ms intervals^3,4,6,11,14,15^. Because of their rich vocal repertoire and their complex calling patterns, bats have been widely used as animal model to address specifics of communication call processing in the central auditory system^3,6,11,16–18^.

In this study, we investigated how natural distress sequences are processed in the auditory cortex (AC) of awake bats. We were interested in unveiling whether multiscale temporal structures present in bat vocalizations could be encoded by auditory cortical neurons. Experimental evidence indicates that electrophysiological oscillations in the human AC can represent different timescales existing in speech sounds, allowing the simultaneous processing of the syllabic rate (at rhythms of 4–8 Hz, consistent with theta-band oscillations), and the phonemic rate (rhythms of >30 Hz, consistent with gamma-band fluctuations)^19–21^. Although such multiscale processing at distinct frequencies is well explored in humans, little is known regarding the representation of different vocalization timescales in other mammals, especially at a neuronal level in the cortex. Here, we aimed to bridge this gap by recording electrophysiological activity from the AC of the bat *Carollia perspicillata* in response to natural distress sequences, comprising at least two different timescales: the syllabic rate and the bout rate. Both the AC and the distress communication repertoire of this species have been well investigated^3,4,22–24^, making it a suitable model for studying the specifics of auditory temporal processing in the brain. Specifically, we pursued answers to the following questions.

First, is the multiscale temporal structure of distress vocalizations represented by cortical neurons, or are phenomena such as neuronal suppression or phase-locking limitations hindering it? These two phenomena affect neuronal responses by preventing activation to sounds presented at fast rates (> 10–30 Hz)^25,26^. In anesthetized bats, they seem to reduce the neuronal capability to track the temporal structure of natural sequences^3,23^; whether the same occurs in awake animals remains unknown.

Second, how is the spiking activity evoked by natural vocalizations related to ongoing local-field potentials (LFPs)? LFPs constitute slow electrophysiological signals that represent the composite activity of a certain brain region^27^, and are important for sensory processing and neuronal computations^28,29^. These potentials correlate with activity measured using non-invasive methods such as electro-encephalogram and functional magnetic resonance imaging^30^. Although spike–LFP coupling has been extensively studied in the visual and somatosensory systems^31–34^, the interactions between these signals in the auditory modality during communication call processing is less clear.

We found that in the AC of *C. perspicillata*, the multiscale temporal structure of distress calls is encoded by three neuronal subpopulations. These neuronal types have different response patterns that represent distinct aspects of the sequences’ temporal features, and each of them is informative about the calls to a different degree. Moreover, the three subpopulations are differently synchronized to ongoing cortical oscillations. Taken together, our results highlight the existence of a multiscale temporal representation of natural vocalizations at a neuronal level in the AC. Such segregation could contribute to a parsed representation of temporal acoustic structures in the mammalian brain.

## Results

### Three main types of neurons coexist in the AC

**Fig. 1:**
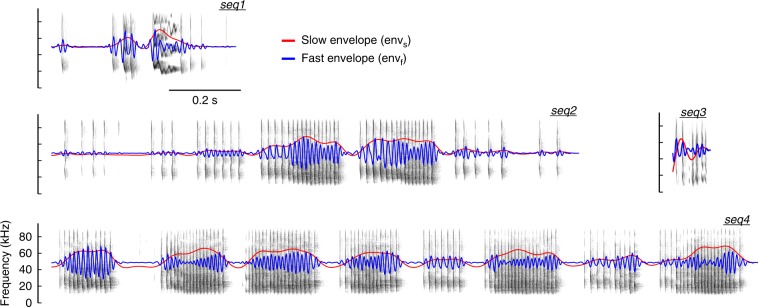
Natural distress calls used as stimuli. Spectrotemporal representation of the four natural distress sequences used as stimuli in the study. The slow (0.1–15 Hz, env_s_) and fast (50–100 Hz, env_f_) amplitude envelopes of each call are shown within the spectrograms in red and blue, respectively

Electrophysiological data from 87 units (i.e. spike-sorted neuronal responses) in the AC of awake bats (*C. perspicillata*; six animals, two males) were recorded in response to four natural distress sequences (seq1, seq2, seq3, and seq4). The spectrotemporal structure of the calls is depicted in Fig. 1, and their general properties summarized in Supplementary Table 1.

**Fig. 2:**
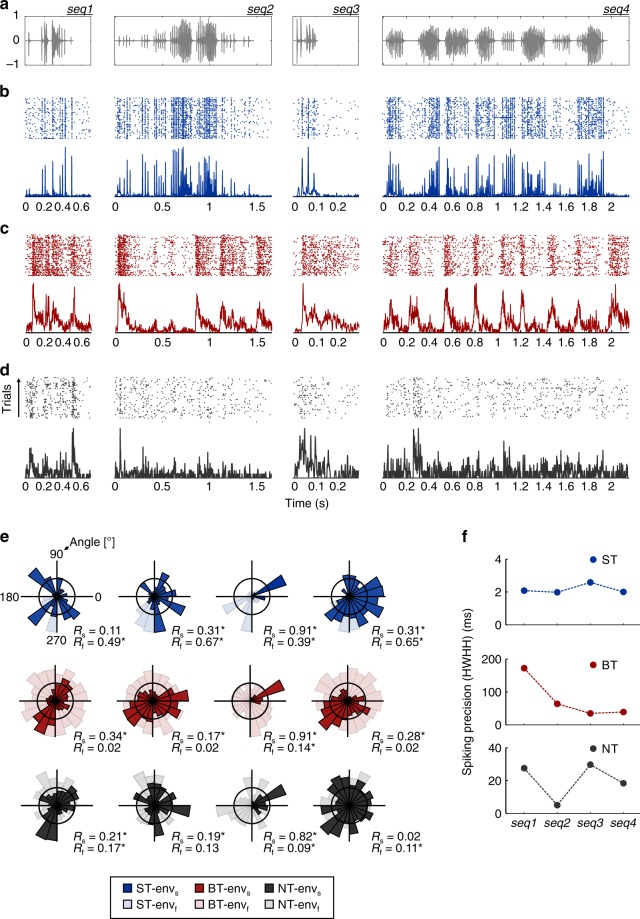
Three types of units coexist in the bat auditory cortex. **a** Oscillogram of the natural distress sequences used as stimuli in this study (see also Fig. 1). **b** Raster plots (top) and spike probability density function over time (bottom; 1 ms precision) of a representative syllable-tracking (ST) unit, responding to each of the distress calls tested. The response to a specific sequence is aligned with panel **a** for clarity. **c** Response of a representative bout-tracking (BT) unit, shown following the same conventions as in **b**. **d** Response of a non-tracking (NT) unit, shown following the same conventions as in **b** and **c**. **e** Circular distribution of spike phases (arranged by calls across columns and unit type across rows; note the color coding for identifying each unit type). Spike phases relative to the slow (0.1–15 Hz) stimulus envelope are shown in dark colors, while spike phases relative to the fast (50–100 Hz) envelope are shown in light colors. **f** Neuronal precision (quantified with the HWHH, see main text) of the three example units, across stimuli. Note the differences in scale of *y*-axes (env_s_: slow enlveope; env_f_: fast envelope; *R*_s_: mean vector, slow envelope; *R*_f:_ mean vector, fast envelope)

Units in the AC of *C. perspicillata* responded differently to the temporal structure of the sequences. A subset of them were able to represent discrete call elements (i.e. individual syllables) in a reliable and precise manner across trials, and were therefore termed syllable-tracking (ST) units (see example in Fig. 2b). Another subset of units, which were unable to reliably track discrete syllables, could represent the slower temporal structure of the calls (i.e. its bouts). As illustrated in Fig. 2c, this ability becomes apparent in response to seq2 and seq4, where the unit’s spiking followed the calls’ bout structure; such units were termed bout-trackers (BT). Thus, ST and BT units represented different timescales in the temporal structure of the sequences: their (fast) syllabic and (slow) bout structures, respectively. Finally, a third subset of units showed no evident tracking abilities to either the bout or syllabic structures of the vocalizations. These units are referred to as non-tracking (NT) units and are exemplified in Fig. 2d.

As a marker of the bout and syllabic temporal structures of the calls, we used the slow (0.1–15 Hz) and fast (50–100 Hz) components of their amplitude envelope (env_s_ and env_f_, respectively; see Fig. 1). Spike times of each unit were related to the instantaneous phase of env_s_ and env_f_, and circular statistics were performed to quantify the synchronization capacity of the spiking to either of them (see Methods). Spike phases relative to the slow and fast envelopes, computed for the ST, BT, and NT units shown in Fig. 2b–d, are depicted in Fig. 2e. To quantify phase-locking strength we used the mean circular vector of the spike phases relative to either env_s_ or env_f_ (*R*_s_ or *R*_f_, respectively). If a unit showed a significant synchronization (Rayleigh test, *p* < 0.001) to the fast envelope in every sequence tested it was classified as ST, whereas if a unit was significantly synchronized to the slow temporal structure of all stimuli, but not to the fast one, it was classified as BT. NT units did not fulfill any of these criteria. In total, we found 22 ST units, 37 BT units, and 28 NT units.

A prominent feature of ST responses was the high across-trial precision of their spiking activity, evidenced by the width of the peaks in their spike probability density function over time (sPDF; Fig. 2b, bottom). Neuronal precision was quantified as the half-width half-height (HWHH) of the main peak of the autocorrelation of a unit’s sPDF^35^, in response to a certain call. Values of HWHH for the example ST, BT, and NT units are shown in Fig. 2f. Although the spiking precision was affected by the call under consideration in the representative BT and NT units, the ST unit’s precision was consistently ~2 ms, and therefore considerably better. Note that while ST, BT, and NT units did not differ significantly in their spike shape (according to their spike width, Supplementary Figure 1), these neuronal groups showed slight differences regarding their frequency tuning (Supplementary Figure 2).

### Differences in temporal response properties across calls

**Fig. 3:**
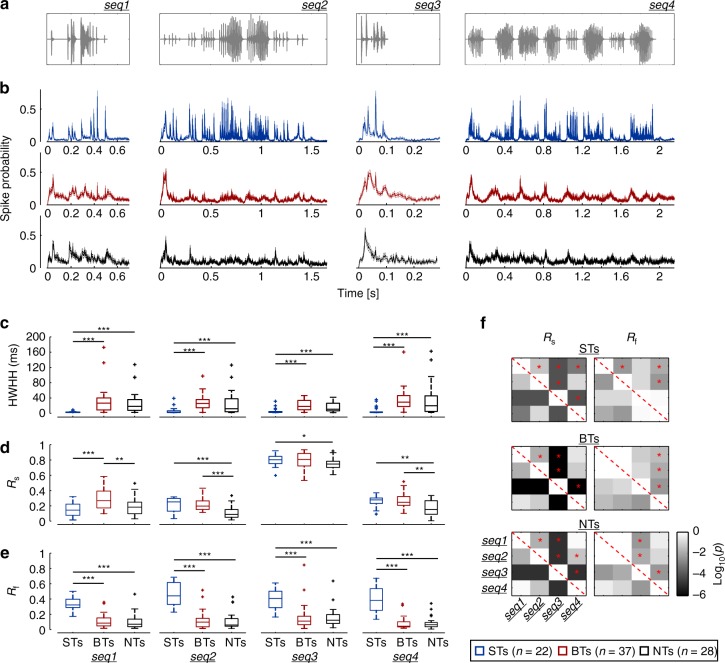
Population response properties across calls. **a** Oscillogram of the natural distress calls used as stimuli. **b** Average population responses of ST (blue), BT (red), and NT (black) units in response to the distress sequences (shown as spike probability density function over time, 1 ms precision; thick line depicts the mean, whereas thinner lines represent the SEM). **c** Neuronal precision (measured as HWHH, see main text) of the three subpopulations in response to the natural stimuli (from seq1 to seq4, left to right). Syllable-tracking units were always significantly more precise than BT and NT units (FDR-corrected Wilcoxon rank-sum tests, *p*_corr_ ≤ 0.0013), but no significant differences occurred between BTs and NTs in any of the calls tested (*p*_corr_ > 0.08). **d** Comparison of vector strength from the spike phases relative to the slow (0.1–15 Hz) call envelopes (*R*_s_). Across sequences and according to their *R*_s_ value, NT units were significantly less synchronized than BT units (*p*_corr_ ≤ 0.02; except in the shortest call, seq3). **e** Comparison of vector strength from spike phases relative to the fast (50–100 Hz) call envelope (*R*_f_). ST units were always better synchronized to the fast temporal structure of the sequences (*p*_corr_ < 2.2 × 10^−6^), while no differences occurred between BT or NT units (*p*_corr_ > 0.45). **f** Significance matrix of statistical comparisons (Wilcoxon signed-rank test, FDR corrected) between vector strength values (*R*_s_, left column; *R*_f_ right column) across calls, for each neuronal type (STs, BTs, or NTs, indicated in the figure). Each cell (*i,j*) in a matrix shows the corrected *p*-value obtained by comparing the *R* value of each group in response to two different sequences, *seq(i)* and *seq(j)*. For example, the red star in cell (*1,2*) of the upper left matrix indicates that when considering the *R*_s_ of ST units, there were significant differences between responses elicited by seq1 and seq2. Note that these matrixes are symmetrical along the diagonal (red dashed line) (*p_corr_ < 0.05; **p_corr_ < 0.01; ***p_corr_ < 0.001)

At a population level, ST, BT, and NT units responded similarly to the examples shown in Fig. 2b–d. Figure 3b shows that ST units followed the syllabic rate of the calls, whereas BT units represented their bout structure, particularly in response to seq2 and seq4 (those with more than one bout). To address differences in the temporal response properties of the three neuronal subtypes (across calls and within groups), we compared the two metrics used to characterize the units’ spiking: their HWHH and *R* values. In terms of spike precision, and regardless of the call under consideration, ST units were always significantly more precise than BT and NT units (Fig. 3c; FDR-corrected Wilcoxon rank-sum tests, corrected *p* (*p*_corr_) ≤ 0.0013; median precision in ST units: 2.7 ms), although BT and NT units did not differ significantly in terms of HWHH (*p*_corr_ > 0.08). While the HWHH values of BT and NT units were affected by the call analyzed (Friedman’s test; BTs: *p* = 0.009; *Χ*^2^ = 11.56; df = 3; NTs: *p* = 0.01; *Χ*^2^ = 10.93; df = 3), post hoc tests revealed no significant pairwise differences for the responses across calls in any group, except for responses of BT units between seq3 and seq4, and of NT units between seq1 and seq3 (FDR-corrected Wilcoxon signed-rank tests; *p*_corr_ < 0.03). Conversely, the precision of ST units was not significantly affected by the sequences (Friedman’s test, *p* = 0.08; *Χ*^2^ = 6.71; df = 3). These results indicate that differences in the spiking precision of ST, BT, and NT units were preserved across calls, and that the HWHH was not consistently affected by the call’s structure.

Considering the synchronization abilities to the slow envelope of the sequences, BT and ST units typically had significantly higher *R*_s_ than NT units (Fig. 3d; FDR-corrected Wilcoxon rank-sum tests, *p*_corr_ ≤ 0.02), except in response to seq1 where groups of ST and NT units did not differ significantly (*p*_corr_ = 0.46), and in response to seq3 (the shortest sequence) where the *R*_s_ of BT and NT groups was not significantly different (*p*_corr_ = 0.065). Regarding the units’ *R*_f_, STs were significantly better synchronized to env_f_ than BTs and NTs, in every call tested (Fig. 3e; FDR-corrected Wilcoxon rank-sum tests, *p*_corr_ < 2.2 × 10^–6^), while the *R*_f_ of BT and NT units was not significantly different across vocalizations (*p*_corr_ > 0.45). How the sequences affected the values of *R*_s_ and *R*_f_ for each neuronal group is illustrated in the significance matrixes shown in Fig. 3f.

These results highlight differences in responses of ST, BT, and NT units, indicating that ST and BT units were able to represent distinct timescales present in the sequences’ envelopes, whereas NT units were altogether poorly locked to the calls. We further tested this notion by evaluating the presence of periodicities in the spiking of each neuronal group, and observed that ST and BT units carried periodicities in their response at fast (50–100 Hz) and slow (0.1–15 Hz) rates, respectively, while NTs did not (Supplementary Figure 3). The former is in accordance to their synchronization abilities to the syllabic or the bout structure of the calls.

### Spike–LFP coherence patterns in the AC are group specific

We addressed whether the differences presented above would also be reflected in the way the spiking activity synchronized to ongoing LFPs. To quantify synchronization, we used the spike–field coherence (SFC) metric^29,34^, a normalized, frequency-dependent index that measures how well spikes lock to LFPs (0 no coherence; 1 perfect coherence). The SFC was calculated only in response to the two longest sequences (seq2 and seq4), and in units that fired at least 150 spikes across all trials of each sequence (14/22 ST, 28/37 BT, and 15/28 NT units, see Methods).

**Fig. 4:**
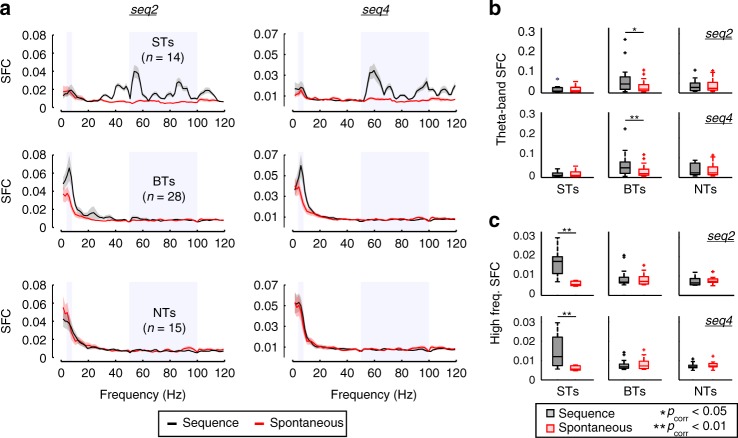
Spike–LFP coherence patterns are group specific. **a** Population spike–field coherence (SFC) for ST (top), BT (middle), and NT (bottom) units, in response to calls seq2 (left column) and seq4 (right; i.e. the two longest calls in this study). Vertical shaded areas indicate frequency bands of 4–8 and 50–100 Hz (that is, theta-band and high- frequency band of the LFP, respectively). Black traces indicate coherence calculated with spikes and LFPs recorded during sound stimulation, whereas red traces show coherence during spontaneous activity (solid lines, mean; shaded areas, SEM). **b** Comparison of average theta-band SFC in response to both sequences (top, seq2; bottom, seq4). Only BTs significantly increased their theta-band spike–LFP synchrony during stimulation as compared to spontaneous coherence (FDR-corrected Wilcoxon signed-rank tests, *p*_corr_ ≤ 0.03 in the case of BT units). **c** Same as in **b** but considering SFC in high frequencies (50–100 Hz). Only ST units significantly increased their high-frequency spike–LFP coherence during acoustic stimulation, in response to either sequence (*p*_corr_ ≤ 0.0024). (*p_corr_ < 0.05; **p_corr_ < 0.01)

Compared to a baseline SFC calculated from spontaneous firing (see Supplementary Figure 4 for a representation of the spontaneous activity), the coherence in each group during sound processing exhibited different trends: ST units increased their spike–LFP synchrony in high frequencies (50–100 Hz) of the spectrum, BT units showed increased synchronization mainly in the theta-band of the spectrum (4–8 Hz), whereas NT units did not differ from spontaneous activity in terms of their SFC (Fig. 4a). The above was true in response to seq2 and seq4, and was corroborated by statistically comparing the mean SFC in theta- and high-frequency bands of the LFPs. Theta-band SFC in BT units was significantly higher during stimulation than during spontaneous activity (Fig. 4b; FDR-corrected Wilcoxon signed-rank tests, *p*_corr_ ≤ 0.03), but was not affected in ST or NT units (*p*_corr_  > 0.8). Additionally, only ST units significantly increased their synchronization to high-frequency LFPs (Fig. 4c; *p*_corr_ ≤ 0.0024 for ST units; *p*_corr_ > 0.37 for BT and NT units). Such spike–LFP coherence in high LFP frequencies is not necessarily related to gamma-band computational processes (see ref. ^28^, and Discussion), but rather to the syllabic rate which entrains both the LFPs and the spiking of ST units. High-frequency LFP-stimulus entrainment has been previously reported in this species^3,36^, and was quantified here using the stimulus–field coherence metric (a frequency-dependent index similar to the SFC; see Supplementary Methods), depicted in Supplementary Figure 5. Overall, the coherence patterns suggest distinct cortical processes underlying the response type of each neuronal subpopulation, particularly implicating low frequency oscillations in the organization of BT-like responses. In addition, although there was phase–amplitude coupling (PAC) between low (4–8 Hz) and high-frequency LFPs (50–100 Hz) in all cases studied, such PAC did not directly account for differences between the ST, BT, and NT spiking (see Supplementary Figure 6).

### Rate-of-fire information across neuronal groups

**Fig. 5:**
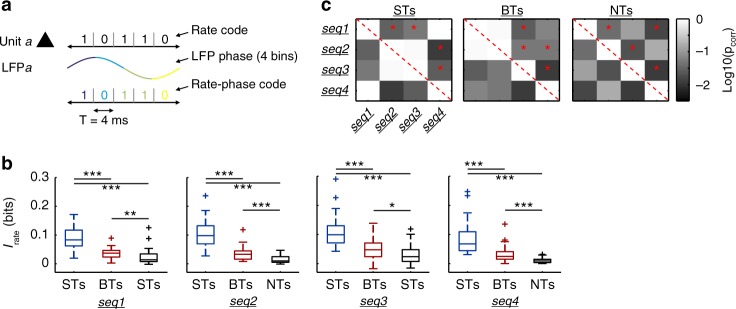
Syllable-tracking units provide the highest information in terms of spiking rate. **a** Representation of the main neuronal codes used in the study: a rate code (*I*_rate_), determined by the neuronal spiking; a phase code (*I*_phase_), determined by binned phases of the LFP; and a rate-phase code (*I*_rate_phase_), which combines both of the above. **b** Information in the rate code of the three neuronal groups (STs, BTs, and NTs; represented in blue, red, and black, respectively). Significance was assessed after FDR-corrected Wilcoxon ranksum tests. Note that ST units were the most informative in terms of firing-rate (*p*_corr_ < 2.6 × 10^−5^). **c** Significance matrixes showing the results of statistically comparing *I*_rate_ for each neuronal group, across sequences. Conventions as defined in Fig. 3f. Briefly, cell (*i,j*) in a matrix represents the *p*-value of comparing *I*_rate_ in response to sequences *i* and *j*, respectively. For example, in the leftmost matrix the star in cell (*1, 2*) indicates that *I*_rate_ in ST units significantly differed in response to *seq1* and *seq2*. Note that the matrixes are symmetrical along the diagonal (red dashed line). (*p_corr_ < 0.05; **p_corr_ < 0.01; ***p_corr_ < 0.001)

Possible differences in the encoding capabilities of ST, BT and NT units were addressed by means of Shannon mutual information (MI; also “information” throughout the manuscript). To quantify the MI between neurophysiological signals and auditory streams, aspects of the responses can be translated into so-called neural codes that approximate plausible representations of the stimuli. In this section, we focus on a neural code defined by the spiking rate (*I*_rate_) of individual units (Fig. 5a, top; see Methods).

Regarding *I*_rate_, the three neuronal groups were informative to different degrees (Fig. 5b). In response to all sequences tested, ST units were most informative, followed by BT and NT ones (FDR-corrected Wilcoxon rank-sum tests; STs vs. BTs, *p*_corr_ < 2.6 × 10^–5^; STs vs. NTs, *p*_corr_ < 6.5 × 10^–7^). Despite being less informative than BT units (*p*_corr_ ≤ 0.013), NT units were still informative to some extent (median of 0.0125 bits, equivalent to 3.12 bits/s), indicating that the slow or fast temporal envelope of the calls do not have to be explicitly and consistently reflected for the spike patterns to carry information about the stimulus. We addressed whether and how the information content varied across calls, in each neuronal subpopulation. Fig. 5c shows significance matrixes summarizing statistical comparisons of *I*_rate_, for ST, BT, and NT units, across sequences. The data suggest that the spectrotemporal structure of the vocalizations altered their coding by cortical spiking.

### Information content of LFP phase

**Fig. 6:**
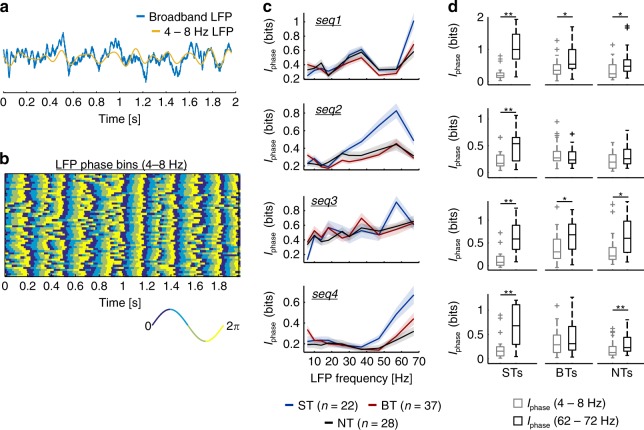
Information content of LFP phase. **a** Single trial broadband (blue) and theta-band (4–8 Hz; yellow) LFP traces. Depicted field potentials were recorded in response to seq4 and correspond to the same exemplary BT unit shown in Fig. 2c. **b** Binned LFP phase across trials of theta-band LFPs, according to the phase discretization used to calculate *I*_phase_. The convention for the bins is shown at the bottom (note that the binning precision was of *π*/2). **c** Information content of the LFP phase (*I*_phase_) associated to ST (blue), BT (red), and NT (black) units, in different frequency bands and across all sequences tested. Data is presented as mean (solid lines) ± SEM (shaded areas). **d** Statistical comparisons (FDR-corrected Wilcoxon signed-rank test) of *I*_phase_ in theta-band LFPs (light gray) vs. *I*_phase_ in high-frequency LFPs (62–72 Hz; black). Each row depicts comparisons for a particular call, considering ST, BT, or NT units (arranged in columns left to right, respectively). (*p_corr_ < 0.05; **p_corr_ < 0.01)

We systematically calculated the MI between the LFP phase and the stimuli (*I*_phase_), in different frequency bands (ranging from 4 to 72 Hz), across neuronal groups. Fig. 5a (middle) illustrates the coding scheme used to obtain *I*_phase_: in this case, information was quantified by binning the LFP phase into four equally sized angular quadrants (see Methods). The lowest LFP band considered (theta, 4–8  Hz) is portrayed together with a broadband LFP trace in Fig. 6a (single trial LFP activity recorded simultaneously to the spiking of the BT unit shown in Fig. 2c, in response to seq4). The binning scheme used to calculate *I*_phase_ values presented in the main results (four bins), for all trials (*n* = 50), is depicted in Fig. 6b for theta-band oscillations.

Patterns of *I*_phase_ at different frequency bands varied depending on the sequence analyzed, suggesting that the temporal structure of the call’s envelope altered the *I*_phase_ values at distinct LFP frequencies (Fig. 6c). In fact, the tendencies of *I*_phase_ were consistent with the trends of LFP phase synchronization observed for each group, across calls (compare *I*_phase_ in Fig. 6c with the LFP–stimulus coherence in Supplementary Figure 5). Note, in Supplementary Figure 5, that LFPs showed strong stimulus-synchrony at frequencies matching the syllabic rate of the calls. Consequently, the information content of the LFP phase did not deteriorate at higher frequency bands (>50 Hz), and fast LFPs, which could entrain to the syllabic rate of the sequences, were at least as informative as low-frequency oscillations. The former was assessed by statistically comparing the information of LFP phase in the 4–8 and 62–72 Hz ranges (the lowest vs. the highest frequency bands studied). Figure 6d shows that *I*_phase_ in high frequencies could be significantly higher than *I*_phase_ in the theta-band (FDR-corrected Wilcoxon signed-rank test, *p*_corr_ < 0.034), and that otherwise they did not differ significantly (*p*_corr_ > 0.3).

### Low- and high-frequency LFPs increase information in spiking

**Fig. 7:**
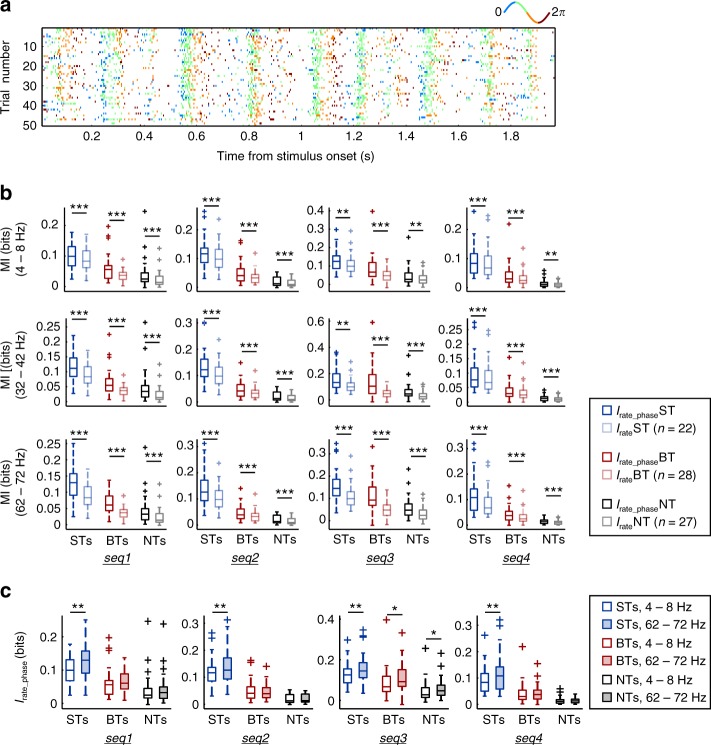
Spike rate information content is increased by the phase of low and high frequency LFPs. **a** Raster plot of the BT unit shown in Fig. 2c (in response to seq4), in which spikes are color-coded according to the phase of 4–8 Hz LFPs. Note the bin color scheme in the panel. **b** Information content of the phase-of-fire code (*I*_rate_phase_) vs. the rate code (*I*_rate_), for each neuronal group (ST, BT, and NT), in three different LFP bands: a low frequency band (4–8 Hz, or theta), a middle frequency band (32–42 Hz), and a high frequency band (62–72 Hz). Comparisons are performed for each natural sequence tested, showing that *I*_rate_phase_ was always significantly higher than *I*_rate_ (FDR-corrected Wilcoxon signed-rank tests, *p*_corr_ ≤ 0.0037), also in the highest frequency band considered, 62–72 Hz. **c** Comparisons between *I*_rate_phase_ obtained using low-frequency LFPs (4–8 Hz), vs. *I*_rate_phase_ using high-frequency oscillations (62–72 Hz), for each neuronal group, in every call tested. *I*_rate_phase_ calculated with 62–72 Hz LFPs was either significantly higher than *I*_rate_phase_ computed with 4–8 Hz LFPs (FDR-corrected Wilcoxon signed-rank tests, *p*_corr_ ≤ 0.026), or at least not significantly different (*p*_corr_ > 0.077). (*p_corr_ <  0.05; **p_corr_ < 0.01; ***p_corr_ < 0.001)

With the aim of addressing if the phase of the LFP in different frequencies contributed additional information to the spiking responses, we quantified the MI considering a joint code that encompassed both the units’ firing rate, and the phase of ongoing oscillations (*I*_rate_phase_; Fig. 5a, bottom). The code was constructed by labeling spikes with the LFP phase, and calculating *I*_rate_phase_ from the phase-labeled spiking (see Methods). A representation of such coding scheme is depicted in Fig. 7a, where spikes were color-coded according to the phase of theta-band LFPs, discretized into four equally sized bins. The quantification of *I*_rate_phase_ was performed in three different LFP bands: 4–8, 32–42, and 62–72 Hz, representing low, middle, and high frequency oscillations.

Independently of the frequency band considered, and in response to each stimulus tested, *I*_rate_phase_ was always significantly higher than *I*_rate_ (Fig. 7b; FDR-corrected Wilcoxon signed-rank tests, *p*_corr_ ≤  0.0037). The former was true for ST, BT, and NT units, and not only implies that a phase-of-fire code increases the information content of the neuronal spiking, but that such increase also occurs taking into account the phase of high-frequency oscillations. These results were striking (see refs ^37,38^), although not unexpected, since the phase of high-frequency LFPs was at least as informative as the phase of theta–band oscillations (Fig. 6d), and because high-frequency LFPs were phase-locked to the calls’ syllabic rate (Supplementary Figure 5). Moreover, high-frequency oscillations yielded, in response to some calls (all of them in the case of ST units), significantly higher *I*_rate_phase_ values than low-frequency LFPs (Fig. 7c; FDR-corrected Wilcoxon signed-rank tests, *p*_corr_ ≤ 0.026). Remarkably, the phase-of-fire information was novel across calls and frequency bands (including the 62–72 Hz range), and not a mere consequence of the neuronal firing rate (see Supplementary Figure 7). Taken together, our data indicate that the information provided by a phase-of-fire code does not necessarily deteriorate for high-frequency oscillations in the AC, provided that such oscillations are also stimulus-informative.

### Paired neuronal responses provide independent information

**Fig. 8:**
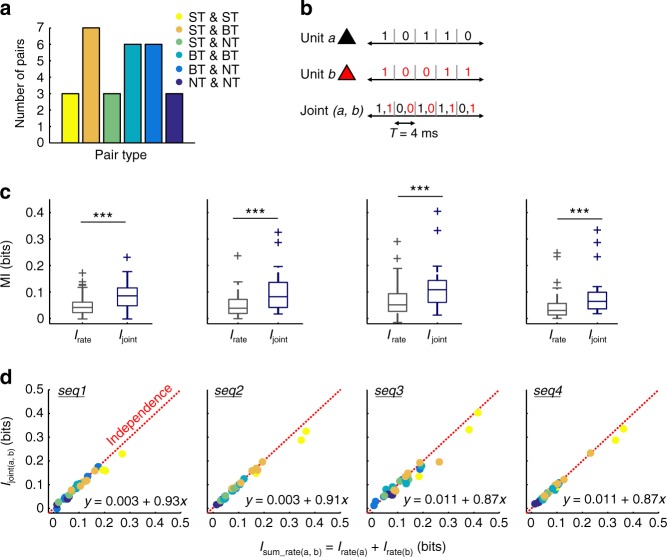
Auditory cortical units provide mostly independent information. **a** Quantification of neuronal pair types according to their composition (based on the unit classification as ST, BT, or NT). **b** Schematic representation of the joint response of two neurons, *a* and *b*, used to quantify *I*_joint_. **c** Statistical comparisons of *I*_joint_ (blue) vs. *I*_rate_ (gray) in each of the natural calls tested. In all cases, *I*_joint_ was significantly higher than *I*_rate_ (FDR-corrected Wilcoxon rank-sum tests, *p*_corr_ < 5.3 × 10^−4^). **d** Information carried by a joint response of a pair (*I*_joint_) plotted vs. the sum of information in the rate code (*I*_rate_) of each unit comprising such pair (each dot represents a pair, color coded according to the units that compose it; see also panel **a**), for each natural sequence tested. The red dashed line represents the regime in which two neuronal responses convey independent information (i.e. *I*_joint_ = *I*_rate(*a*)_ + *I*_rate(*b*),_ with a slope of 1). Points below the line represent redundant interactions, whereas points above the line represent synergistic interactions between units in a pair. Regression slopes for observed data are indicated in each panel. (***p_corr_ < 0.01)

Twenty-eight responses were simultaneously recorded from pairs of units (56 units) in *C. perspicillata*’s AC. A histogram of unit pair composition, in terms of their classification as STs, BTs, or NTs, is given in Fig. 8a. The units in each pair were at least 500 µm apart in the cortical surface. From these paired responses, we calculated the information by means of a neural code which represented the nested activity of two units (*I*_joint_). In terms of response quantification, *I*_joint_ measures how much information is provided by the spiking of both units, taking into account the identity of the unit that fired (or not) a spike (Fig. 8b).

Simultaneously recorded responses from two units in the AC were overall more informative about the calls than unpaired neuronal spiking. That is, *I*_joint_ was, across sequences, significantly higher than *I*_rate_ (Fig. 8c; FDR-corrected Wilcoxon rank-sum tests, *p*_corr_ < 5.3 × 10^–4^). Therefore, possible interactions between at least two neurons can significantly reduce the uncertainty about the stimulus, such that a putative higher-order structure, or post-synaptic neuron, benefits from such interactions for sensory coding. The former leads to the question of how paired responses could contribute to the processing of the calls. Three possibilities arise: (i) the joint information of units *a* and *b* in a pair is less than the sum of information provided by each unit (i.e. *I*_joint(*a*,*b*)_ < *I*_sum_rate(*a*,*b*)_; where *I*_sum_rate(*a*,*b*)_ = *I*_rate(*a*)_ + *I*_rate(*b*)_), implying that both responses carry redundant information; (ii) the joint information amounts to the sum of information provided by each unit (i.e. *I*_joint(*a*,*b*)_ = *I*_sum_rate(*a*,*b*)_), implying that both responses carry independent information; or (iii) the joint information is higher than the sum of information provided by the units (i.e. *I*_joint(*a*,*b*)_ > *I*_sum_rate(*a*,*b*)_), implying that both responses interact synergistically. Paired units in the AC provided mostly independent information across calls (Fig. 8d). In the figure, values of *I*_joint(*a*,*b*)_ are plotted against values of *I*_sum_rate(*a*,*b*)_, and points in the scatter plots represent neuronal pairs, color-coded according to the types of units that comprise them. Note that such points lie tightly close to the regime of independence (*I*_joint(*a*,*b*)_ = *I*_sum_rate(*a*,*b*)_), depicted with red dashed lines (slope of 1; regression slopes for the data are indicated in the panels). However, *I*_joint(*a*,*b*)_ was, albeit to a small degree, significantly lower than *I*_sum_rate(*a*,*b*)_ for every call except seq4 (FDR-corrected Wilcoxon signed-rank test; *p*_corr_ < 0.05; *p*_corr_ = 0.0502 in the case of seq4). This suggested slight redundancies in the responses and could represent an effect of analyzing pairs composed of the same type of units (e.g. ST–ST pairs). In fact, when taking into account only pairs consisting of different types of units (i.e. ST–BT, ST–NT, and BT–NT pairs; *n* = 16), significant differences between *I*_joint(*a*,*b*)_ and *I*_sum_rate(*a*,*b*)_ were abolished across sequences (FDR-corrected Wilcoxon signed-rank tests, *p*_corr_ > 0.07). Altogether, these results suggest that auditory cortical units provide independent information about the calls used as stimuli, and support the reported functional segregation in the representation of temporal features in the AC.

## Discussion

In this study, we investigated the relationship between neuronal responses and the multiscale temporal structure of bat distress vocalizations, manifested mainly at two different timescales: the syllabic and the bout repetition rates. We report five main findings: (i) different units in the AC represent different levels of the temporal structure of natural calls (the syllabic or the bout rates of the sequences); (ii) neuronal spiking synchronizes differently to ongoing LFPs, depending on which temporal features of the natural sequences the units are able to encode; (iii) the information content in spiking rate depends on which timescale of the sequences is encoded by the units; (iv) the phase of low and high frequency LFPs is informative about the perceived acoustic streams; and (v) paired neuronal responses in the AC convey independent information, in consonance with the observed functional segregation.

Electrophysiological recordings from the AC of awake *C. perspicillata* revealed the existence of three groups of units, two of which represented different levels of the multiscale temporal structure of the natural sequences: syllable-tracking, bout-tracking, and non-tracking units (Fig. 2b–d). Sustained representations of natural acoustic streams, such as those recorded from BT or ST units, were not observed in previous studies addressing the processing of natural vocalizations in *C. perspicillata*’s AC^3,23,39,40^. In those cases, in which the animals were ketamine-anesthetized, cortical suppression hindered AC neurons from eliciting sustained BT-like or, more noticeably, ST-like responses. The former could be explained by the fact that ketamine anesthesia affects the response of auditory neurons, often by inducing suppression^41,42^. Moreover, we show the existence of synchronized responses to the fine temporal structure of natural distress sequences, where syllables are typically repeated at rates higher than 50 Hz. To our knowledge, this is the first report of neurons in the bat AC that follow the fast-varying structure of communication calls. Although in bats and other mammals cortical units can usually only track relatively slow (<20–30 Hz) amplitude modulated sounds in a phase-locked manner^23,26,43,44^, the presence of embedded modulations in acoustic stimuli could enhance their temporal representation in the cortex^45^. Two potential mechanisms might account for the above-mentioned enhancement (see ref. ^45^). The first mechanism is based on the depressive character of excitatory thalamocortical inputs (e.g. due to the exhaustion of neurotransmitter at the synapse in the AC). This (synaptic) depression may be attenuated if the fast stimulus is transiently reduced by means of a slower modulatory envelope. The second mechanism involves the slow stimulus envelope modulating the relative timing of inhibition/excitation in a local cortical circuit, which may reduce suppressive effects in the response to fast periodicities. Although our data do not allow to disentangle whether ST responses occur due to specific statistics present in natural vocalizations, embedded temporal modulations are certainly a feature of *C. perspicillata*’s distress calls, since they include both fast and slow temporal dynamics. We speculate that ST units could profit from such entangled modulations for the representation of the syllabic rate.

**Fig. 9:**
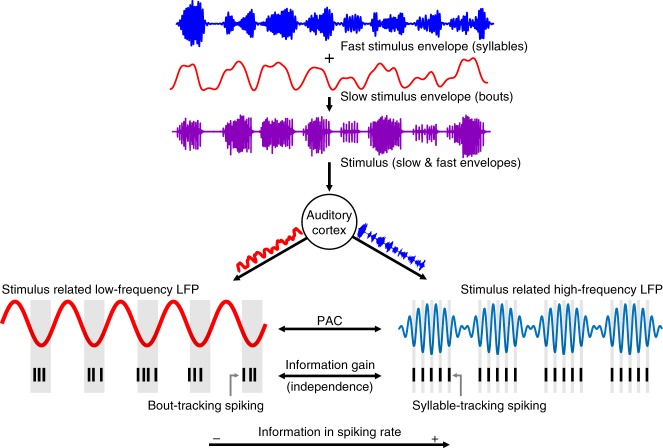
Neuronal processing of multiscale temporal features in the AC. Conspecific distress vocalizations of *C. perspicillata* are typically composed of two embedded temporal scales: a fast one (>50 Hz), consistent with the syllabic rate of the sequence, and a slow one (<15 Hz), consistent with its bout rate. In the AC, such rhythms are represented not only via stimulus-related neuronal oscillations, but also through the spiking patterns of two main neuronal subpopulations: syllable-tracking and bout-tracking units. These subgroups phase-lock to cortical LFPs in distinct frequency bands, in accordance to the temporal features of the calls that they represent (i.e. BTs synchronize to theta oscillations, whereas STs synchronize to LFP frequencies of >50 Hz). While ST units were overall more informative than their BT counterparts, neuronal groups in the auditory cortex which represent distinct timescales present in natural stimuli provided independent information, potentially allowing for a precise and non-redundant encoding, at a neuronal level, of the multiple timescales existent in communication signals

Other studies, particularly in the guinea pig, have reported spiking responses in which the temporal structure of communication calls is well-preserved^9,46,47^. Such responses are called “isomorphic”, since the spiking and the stimulus have similar shape over time. Here, we distinguish between two complementary types of isomorphic responses: BT-like and ST-like spiking. BT and ST units emerge as neuronal correlates of the representation of conspecific vocalizations at different temporal scales. Multiscale processing of communication signals is considered a major feature of the AC, particularly for the representation of speech in humans by cortical oscillations^7,8,21,48^. Our data provide evidence of multiscale temporal processing at a neuronal level in response to communication calls. This implies that the AC (at least in the case of *C. perspicillata*) does not solely rely on oscillations to represent nested temporal modulations present in natural sounds, but also that such features can be tracked by the neuronal spiking. Additionally, based on how syllable- and bout-tracking units synchronize to cortical LFPs (see Fig. 4 and below), the data suggest that BT and ST responses could play a major role in rhythmic networks tasked with de-multiplexing entangled temporal rhythms within complex acoustic stimuli (see ref. ^48^), and might provide a neuronal basis for such computations in the AC. The above is further complemented by the fact that, from a theoretical standpoint, simultaneously recorded units provide mostly independent information (see Fig. 8 and ref. ^37^), suggesting that their interactions provide precise and non-redundant information about auditory stimuli at multiple timescales. These observations are summarized in Fig. 9.

Units with dissimilar temporal response patterns differed regarding their SFC (Fig. 4). For example, while coherence in the theta range (4–8 Hz) increased for BTs during sound processing, the same was not observed in NT or ST units. This suggests that BT units participate in cortical processes that may contribute to the representation of the stimulus’ slow periodicity, mediated by low-frequency (theta-band) LFPs (see refs ^36,49^). Low-frequency neuronal oscillations entrain to the temporal structure of periodic (or even quasi-periodic) sounds^19,50–52^, even allowing in humans the discrimination of speech on a single trial basis^53^. Furthermore, slow fluctuations in LFPs are thought to modulate neuronal responses, ensuring that phases of maximum excitability occur in-time with relevant or predicted stimuli (here, the onset or offset of a bout)^54^. Our results align with the above-discussed views and indicate that BT activity relates to theta-coherent spiking in the AC, supporting that theta oscillations are important for the cortical representation of slow temporal structures at a neuronal level.

Conversely, coherence between spikes and high-frequency (50–100 Hz) LFPs increased significantly only in the case of ST units. Based on the fact that LFPs entrain to the fast-varying syllabic structure of the sequences (see Supplementary Figure 5 and ref. ^3^), and because the spiking in this particular subpopulation is also locked to the syllabic rate, we believe that the increase in high-frequency SFC is due to a simultaneous entrainment of spikes and LFPs, rather than due to traditional gamma-related computational processes, more prominent in the visual modality^28^. Similar oscillatory entrainment to phasic stimuli beyond the spiking synchronization ability has been observed in humans in response to speech and AM sounds^40,55^. The origins of fast oscillations are deemed mostly subcortical^55^, which could be caused by ‘lower’ auditory structures (e.g. the inferior colliculus or the thalamus) being able to more easily represent fast-varying stimuli than the AC^40,56,57^. Alternatively, synchronized high- frequency LFPs could be explained by phase-locked fluctuations in the membrane potentials of cortical neurons^58^, taking into account that subthreshold potentials also shape LFP signals^27^.

Considering the above, we speculate that ST, BT, and NT units may be involved in distinct population dynamics during the processing of vocalization sequences. Coherence results suggest that BT-like responses are mediated by processes involving low-frequency oscillations, which might explain their matched periodicity with the slow temporal structure of the sequences, while ST units may be driven by subcortical synaptic inputs into the AC and could reflect a preservation of precise isomorphic responses in high stages of the auditory pathway.

We quantified the information carried by the three subpopulations in terms of four main neuronal codes that include (and combine) two main features of the measured responses: the spike rate, and the LFP phase^37^. In terms of spiking rate, ST units constituted the most informative subpopulation and NTs were the least informative one. These results suggest that ST units could play a major role in the parsing of auditory stimuli, although the relevance of being able to represent fine aspects of the sequence’s syllabic structure needs to be addressed in future studies. The present data do not allow to properly evaluate whether the syntax of the calls (i.e. the importance of the specific order or timing of its elements^6^) is behaviorally relevant to *C. perspicillata*, and if more or less informative units like STs, BTs, or NTs, play a role in deciphering it. Addressing this questions might prove worthy, bearing in mind that syntactic composition or comprehension are not exclusive to humans^59^, and have also been described in bats^6^.

Previous studies in the visual and auditory systems reported a considerable decrease of information provided by the phase of high-frequency LFPs^37,38,60^. Our data, however, indicate that the phase of high frequency (62–72 Hz) oscillations can be at least as informative as the phase of low-frequency (4–8 Hz) ones (Fig. 6d). We believe that differences between the results reported here and the aforementioned studies could depend mostly on the stimulus’ temporal structure, or on the ability of the system to entrain to fast varying acoustic streams, as it occurs in *C. perspicillata*’s AC (see Supplementary Figure 5, and refs ^3,36^). Nonetheless, the present results highlight the importance of considering the phase of high-frequency oscillations in the AC for neuronal processing, and unveil fast LFPs as a candidate for enhancing the accurate representation of temporal features in naturalistic acoustic streams.

## Methods

### Surgical procedures and animal preparation

All experimental procedures were in compliance with current German laws on animal experimentation and were approved by the Regierungspräsidium Darmstadt (experimental permit #FU-1126). The study was performed on seven adult fruit eating bats *C. perspicillata* (three males). Data from one of the male bats was excluded due to motion artefacts. Animals were obtained from the colony in the Institute for Cell Biology and Neuroscience of the Goethe University in Frankfurt am Main, Germany.

Before surgical procedures, the bats were anesthetized with a mixture of ketamine (10 mg kg^−1^ Ketavet, Pfizer) and xylazine (38 mg kg^−1^ Rompun, Bayer). Local anesthesia (ropivacaine hydrochloride, 2 mg/ml, Fresenius Kabi, Germany) was subcutaneously applied in the scalp prior to surgery, and before any subsequent handling of the wounds. A longitudinal midline incision was made in the skin covering the superior part of the head. Both skin and muscle tissues were carefully removed in order to expose the skull around the AC, in a way that sufficient area of the bone remained uncovered to place a custom-made metal rod (1 cm in length, 0.1 cm in diameter), used to fix the animal’s head during neuronal recordings. The rod was attached to the skull using dental cement (Paladur, Heraeus Kulzer GmbH, Germany).

The location of the AC was assessed macroscopically with the aid of well-described landmarks^3,24^. Once located, the AC was exposed by cutting a small hole in the skull (ca. 1 mm^2^) with a scalpel blade. After surgery, the animals were allowed to rest for at least 2 days before undergoing electrophysiological measurements.

Recordings were performed chronically in fully awake bats in sessions that lasted no more than 4 h a day. Water was offered to the animals during recordings, at periods of ~1–1.5 h. Bats were able to recover for at least one whole day between sessions, and none of them was used more than six times altogether.

### Electrophysiological recordings

All experiments were conducted inside a sound-proofed and electrically isolated chamber, containing a custom-made holder where the bat was placed during the measurements. The temperature of the holder was kept constant at 30 °C by means of a heating blanket (Harvard, Homeothermic blanket control unit). The speaker (NeoCD 1.0 Ribbon Tweeter; Fountek Electronics, China) used for free-field stimulation was located inside of the chamber, 12 cm away from the bat’s right ear (contralateral to the AC of the left hemisphere were recordings were performed), and was calibrated using a $\frac{1}{4}$-inch microphone (Brüel & Kjaer, model 4135, Denmark) connected to a custom-made microphone amplifier.

Electrophysiological data were acquired by inserting two carbon electrodes (Carbostar-1, Kation scientific; Impedance at 1 kHz: 0.4–1.2 M${\mathrm{\Omega }}$) into the AC of the left hemisphere of the bat. The electrodes were separated by at least 500 µm. Two silver wires, touching the dura of non-auditory areas in the contralateral hemisphere, and separated by at least 1 mm, were used as reference electrodes. Such two-reference configuration is useful to minimize issues arousing from common referencing. The depths of the carbon electrodes were accurately controlled from outside of the chamber using two Piezo manipulators (PM-101, Science products GmbH, Hofheim, Germany), one for each electrode. Recordings were performed at depths of 300–500 µm from the cortical surface, corresponding to layers III/IV of the primary AC, although we cannot discard the presence of units obtained from high-frequency fields (see ref. ^24^). Electrical signals were amplified (Dagan EX4-400 Quad Differential Amplifier, Minneapolis, MN; gain = 100, filter low cutoff frequency = 0.01 Hz, high cutoff frequency = 3 kHz), and analog-to-digital converted (RP2.1 Enhanced real time processor, Tucker-Davies Technologies, 2 channel A/D converter, 2 channel D/A converter, 24-bit precision, Alachua, FL) with a sampling frequency of 12.2 kHz, before being sent to the recording computer via USB. The data were stored and monitored on-line with a custom-written Matlab (version 7.9.0.529 (R2009b), MathWorks, Natick, MA) software.

### Acoustic stimulation

The acoustic stimulation was controlled using the same custom-written Matlab software used for data acquisition. Frequency level receptive fields of recorded units were calculated from responses to pure-tone stimuli (10 ms duration, 0.5 ms rise/fall time), with frequencies in the range of 5–90 kHz (5  kHz step), and sound-pressure levels (SPL) in the range of 20–80 dB SPL (10 dB steps). The SPL of the tone was adjusted on-line according to the calibration curve of the speaker. Each frequency-level combination was randomly presented 5–8 times, with an inter-stimulus interval of 250 ms.

We used four distress calls from *C. perspicillata* as acoustic stimuli. Procedures used for recording the vocalizations have been described in detail in a previous study^4^. The four distress sequences used as stimuli are referred to as seq1–seq4 throughout the text, and their spectrotemporal structure is shown in Fig. 1, while Supplementary Table 1 summarizes their main properties. For stimulation, sounds were converted to analog signals with a sound card (M2Tech Hi-face DAC, 384 kHz, 32 bit) and sent to an analog power amplifier (Rotel power amplifier, model RB-1050) in order to be presented through the speaker (see above for specifications) located inside of the chamber. Distress calls used in this paradigm were randomly presented 50 times each. Prior to presentation, the sequences were low-pass filtered (80 kHz cutoff) and were played through the sound card with a sampling rate of 192  kHz. The sequences were multiplied at the beginning and at the end times a linear fading window (10  ms in length, varying from 0 to 1) to avoid artefacts, and were presented with an inter-stimulus interval of 1 s and a pre-time (recording time prior to the onset of the acoustic stimulus) of 10 ms.

Before stimulating with distress sequences, we recorded 50 s of spontaneous activity of the same unit and used these recordings as individual baseline for further analyses.

### Detection of spikes and LFPs

All data analyses were performed off-line using custom-written Matlab (version 8.6.0.267246 (R2015b)) scripts. Spikes and local-field potentials were separated by bandpass filtering the acquired raw electrophysiological signal (fourth-order Butterworth filter, cutoff frequencies: 0.1–300 for LFPs, 300–3000 Hz for spikes). Spike detection was based on their amplitude relative to the recording noise baseline. Specifically, the spike threshold was defined in two steps. First, an initial threshold was calculated using the *z*-score of the electrophysiological trace in each trial. This initial threshold was set to *z* = −3.5 (that is 3.5 standard deviations from the average noise floor). Then, in a second step, the spike detection was manually revised for each neuron to ensure proper detection of spiking activity. Peaks in the signal higher than the defined threshold, and distanced by at least 2 ms, were considered as action potentials (APs). Furthermore, we conducted spike sorting for each recording channel, using the three principal components of the spike waveforms (per channel), and feeding them to the automatic clustering algorithm KlustaKwik^61,62^. Only the most numerous spike cluster was considered for further analyses. Throughout the manuscript, the term ‘unit’ refers to spike-sorted responses.

### De-spiking of LFPs

To reduce spike bleed-through artifacts^63^ when analyzing synchronization between spikes and LFPs, we subtracted from the raw electrophysiological data the median spike waveform at each spike position, taking into account different spike clusters separately (i.e. for each cluster, only its median waveform was subtracted from the raw signal at the specific positions where spikes from this cluster occurred). The de-spiked LFP was then obtained by bandpass filtering the de-spiked raw signal (fourth-order Butterworth filter) between 0.1 and 300 Hz.

### Neuronal precision and synchronization ability

To evaluate the precision of the neuronal responses, we calculated the kernel-smoothened probability distribution across time points of spike occurrences (sPDF), with a 1 ms bandwidth smoothening window. Note that the sPDF is similar to computing a smoothened peri-stimulus time histogram, with 1 ms bins. Following^35,64^, we estimated the spiking precision of each unit (in response to each sequence) by considering the width of the central peak of the autocorrelation function of the unit’s sPDF in response to the stimulus. As a precision measure, we then used the HWHH of the autocorrelograms^35^.

The neuronal synchronization to the temporal structure of the stimulus was calculated considering both the slow (0.1–15 Hz) and the fast (50–100 Hz) envelopes of each sequence (envelopes are shown in Fig. 1 as red and blue traces, respectively). The slow envelope represents temporal dynamics consistent with the bout structure of the calls (particularly in the cases of seq2 and seq4, see Fig. 1), whereas the fast envelope represents temporal dynamics related to the syllabic structure of the sequences. Spike times of a particular unit were expressed relative to the instantaneous phase of the envelopes (slow or fast) at the time of spike occurrence. The resulting spike phases were used to quantify the synchronization of a unit’s spiking to the slow or fast temporal structure of the sequence, by means of circular statistics. We considered a unit to be well synchronized to the slow or fast envelope if its response was significantly phase-locked to the temporal structure of either of them (Rayleigh test, significance after *p* < 0.001). As a measure of phase-locking ability, we used the vector strength (*R*) metric^65^, which is equivalent to the norm of the circular mean vector and is expressed as follows:

1 $$R = \left| {\frac{1}{n}\mathop {\sum }\limits_{k = 1}^n {\mathrm {e}}^{i\phi _k}} \right|$$

where *R* is the vector strength, *n* is the number of spikes elicited in response to the call under consideration, and $\phi _k$ is the phase of the *k*th spike relative to either the slow or the fast envelope.

Considering the above, a unit was defined as ST if its spiking response was significantly synchronized to the fast envelope of the stimulus in response to every sequence tested. Conversely, a unit was defined as BT if its response significantly synchronized to the slow temporal envelope of every call, but not to the fast envelope. NT units were those that did not fulfill any of the above criteria, and thus showed no consistent phase-locking in their spiking to the temporal structure of the stimulus at the two scales (0.1–15 or 50–100 Hz) considered. All circular statistics were performed with the Circular Statistics Toolbox (CircStat)^66^.

### Spike–LFP coherence

We quantified the synchronization between spikes and LFPs using the spike–field coherence (SFC) metric^29,34^. In brief, the SFC is a normalized frequency-dependent coherence index, which ranges from 0 to 1 indicating the strength of the synchronization between spikes and ongoing local oscillations (0, no coherence; 1 perfect coherence). The method is based on the selection of LFP windows, centered at spike times, from a longer LFP trace (note that for SFC analyses, LFPs were previously de-spiked; see above). The LFP segments were averaged and, as a result, the spike-triggered average (STA) was obtained. Because of the averaging procedure, the STA retains only those oscillatory components that are phase consistent across LFP segments, and thus phase consistent with the spike timings. To obtain the SFC, the power of the STA is normalized by the average of the power of each individual LFP window analyzed, which is known as the spike-triggered power (STP). The result of the division is normalized between 0 and 1 and is a good measure to quantify the coherence depending on frequency. Mathematically, the SFC is expressed as follows:

2 $${\mathrm {SFC}}\left( f \right) = \frac{{{\mathrm{\Psi }}({\mathrm{STA}})}}{{\frac{1}{n}\mathop {\sum }\nolimits_{i = 1}^n {\mathrm{\Psi }}(w_i)}}$$

where ${\mathrm{\Psi }}( \cdot )$ is the function used for obtaining the power spectrum, and the denominator is the STP of a series of LFP windows $w_{1,2,3, \ldots ,n}$. The spectrum obtained when calculating coherence values is also referred to as the coherence spectrum.

The SFC is biased by the number of spikes used for its calculation^67^. Therefore, we used only units that elicited at least 150 spikes in response to the sequences being considered and also during spontaneous activity recordings (see above). Since it was unlikely that a unit fired the required number of spikes in a single trial, for each unit, 150 spikes were randomly selected across trials and their associated LFP windows (480 ms in length) were used to quantify coherence. Note that because the window length was relatively large in comparison to the lengths of seq1 or seq3, coherence estimates were only calculated in response to sequences 2 and 4. Spikes used for SFC analyses were randomly selected a total of 500 times, aiming to reduce sampling biases. From the obtained distribution of coherence spectra, we selected the median and considered this value as the ‘true’ SFC of each unit. A similar selection procedure was used for spikes recorded during spontaneous activity, and the resulting SFC (also ‘spontaneous SFC’ throughout the manuscript) was used as a baseline for comparisons. All power spectra were computed using the multitaper method^68^ available in the Chronux toolbox^69^, using two tapers with a time-bandwidth (TW) product of 2.

### Information theoretic analyses

To quantify how much information the neuronal responses carried about the presented stimuli, we used Shannon’s MI (also referred to as ‘information’, *I*(*S*; *R*)) between stimulus and response. In brief, the MI between a stimulus set *S* and a response set *R* can be expressed as follows^70,71^:

3 $$I\left( {R;S} \right) = H\left( R \right) - H\left( {R{\mathrm{|}}S} \right)$$

where *H*(*R*) is the response entropy (i.e. the total variability of the response set) and is calculated as

4 $$H(R) = - \mathop {\sum }\limits_{r \in R} P\left( r \right){\mathrm{log}}_2[P(r)]$$

while

5 $$H\left( {R|S} \right) = - \mathop {\sum }\limits_{s \in S} P(s)\mathop {\sum }\limits_{r \in R} P\left( {r{\mathrm{|}}s} \right)\log _2[P(r|s)]$$

is known as the ‘noise entropy’ and represents the irreproducibility of the response given a stimulus. The probabilities *P*(*r*) and *P*(*s*) represent the probability of a particular response in *R*, and the probability of a particular stimulus in *S*, respectively, while *P*(*r|s*) represents the conditional probability of a response *r* given a stimulus *s*. Both *P*(*r*) and *P*(*s*) depend on the assumptions made regarding how the response is quantified, and how the stimulus set is defined. Particularly, assumptions made on the set of responses *R* are directly dependent on the assumptions made regarding which neural code is to be considered (see below). Note that the units of MI are *bits*, given that the logarithm used for the calculations is of base 2. Each bit of information implies that an observer can reduce its uncertainty about the stimulus (based on the response) by a factor of 2, on a single trial basis.

Following previous studies^37,38^, we quantified the information provided by four main neuronal codes: the rate code (*I*_rate_) of the spiking activity, the phase code (*I*_phase_) of the LFPs, the joint rate–phase code (*I*_rate_phase_), as well as the information carried by the rate code of simultaneously recorded units (*I*_joint_). The characterization of both the stimulus and the response (for each neural code) has been thoroughly addressed in the aforementioned studies and is presented in detail in the Supplementary Methods of this manuscript. All information analyses were conducted using the Information Breakdown Toolbox (ibTB)^72^.

### Estimating information from limited samples

Quantifying the neuronal responses allows to estimate empirical values of *P*(*r*), which can be directly used in equations (4) and (5) using the so-called “direct method”^73^. In the direct method, the quantities calculated with such estimated probabilities will be biased, since it is extremely difficult in practice to observe all possible responses in *R* a sufficiently large number of times, and thus reliably map its probability distribution^74,75^. In our study, we used the quadratic extrapolation (QE) procedure^74^, implemented in the ibTB toolbox, to tackle this bias. Additional to the QE corrections performed, we subtracted any remaining bias by means of a bootstrap procedure described in ref. ^38^. In brief, for the information in the spike-rate and the LFP phase codes (*I*_rate_ and *I*_phase_), bootstraped information values were calculated after pairing at random the response (i.e. spike counts or LFP binned instantaneous phase) with the stimulus, a large number of times (100 repetitions). The above yields a bootstrap distribution of non-zero values if the number of available trials is finite; we then subtracted the median of this distribution from the original estimations of *I*_rate_ and *I*_phase_. For paired response sets (such as those used to calculate *I*_joint_) we also applied the Shuffling procedure implemented, together with the bootstrapping methodology, in the ibTB. Similarly, for the rate-of-fire code, we followed the bootstrapping procedure detailed in ref. ^38^, which effectively destroys the information in phase-of-fire codes (*I*_rate_phase_), but leaves intact the information provided by rate codes (*I*_rate_). This was achieved by pairing the unaltered spiking with randomized binned LFP phase values, which were observed together with the occurrence of a spike (200 repetitions). Note that for all information calculations (including those generating surrogate distributions, or those performed with synthetic data) the QE procedure was applied.

We tested the performance of the bias correction procedures on synthetic data with statistics similar to those of the real data. For rate codes, we generated spike responses (inhomogeneous Poisson processes) which had the same PSTH as each individual unit from our recordings. For phase codes and phase-labeled codes (that is, *I*_phase_ and *I*_rate_phase_, respectively), we simulated binned phase response matrixes with the same probability distribution (per sub-stimulus in the sequence) than the observed in the real data (see also ref. ^38^), and paired them (in the case of *I*_rate_phase_) with the synthetic spike data. Similar approaches have been applied successfully in the past to test for the validity of information-theoretic calculations^35,37,38,60,72^. Supplementary Figure 8 illustrates the performance of our computations with different number of trials per stimulus. The figure indicates that, although different codes suffered differently from biases up to ~16 to 32 trials, the number of trials used in this study (50) was sufficient to estimate robustly the information present in the neural codes here tested, and adequate to support our main conclusions.

### Statistical analyses

Statistical analyses were performed with custom-written Matlab (version 8.6.0.267246 (R2015b)) scripts, using the commercially available Statistics and Machine Learning Toolbox, or other open access toolboxes mentioned accordingly. Multiple comparisons were corrected with the false discovery rate (FDR) approach, using the Benjamini and Hochberg procedure^76^. When appropriate (unless stated otherwise), statistical significance was assessed via nonparametric Wilcoxon rank-sum tests for unpaired data (e.g. when comparing between groups of ST, BT, or NT unis), or Wilcoxon signed-rank tests for paired data (i.e. comparisons between values obtained from the same neuronal group). The significance threshold was set at an alpha of 0.05. Note that, throughout the text, *p* values are reported together with the statistical test conducted to obtain them and were always corrected for multiple comparisons.

## Electronic supplementary material

Supplementary Materials

## Data Availability

The data that support the findings of this study are available from the corresponding authors upon reasonable request.

## References

[1] Seyfarth RM, Cheney DL (2010). Production, usage, and comprehension in animal vocalizations. Brain Lang..

[2] Kanwal JS, Rauschecker JP (2007). Auditory cortex of bats and primates: managing species-specific calls for social communication. Front. Biosci..

[3] Hechavarria JC, Beetz MJ, Macias S, Kossl M (2016). Vocal sequences suppress spiking in the bat auditory cortex while evoking concomitant steady-state local field potentials. Sci. Rep..

[4] Hechavarria JC, Beetz MJ, Macias S, Kossl M (2016). Distress vocalization sequences broadcasted by bats carry redundant information. J. Comp. Physiol. A.

[5] Brudzynski SM (2013). Ethotransmission: communication of emotional states through ultrasonic vocalization in rats. Curr. Opin. Neurobiol..

[6] Esser KH, Condon CJ, Suga N, Kanwal JS (1997). Syntax processing by auditory cortical neurons in the FM-FM area of the mustached bat *Pteronotus parnellii*. Proc. Natl Acad. Sci. USA.

[7] Luo H, Poeppel D (2012). Cortical oscillations in auditory perception and speech: evidence for two temporal windows in human auditory cortex. Front. Psychol..

[8] Poeppel D (2003). The analysis of speech in different temporal integration windows: cerebral lateralization as 'asymmetric sampling in time'. Speech Commun..

[9] Wallace MN, Grimsley JM, Anderson LA, Palmer AR (2013). Representation of individual elements of a complex call sequence in primary auditory cortex. Front. Syst. Neurosci..

[10] Wohlgemuth MJ, Sober SJ, Brainard MS (2010). Linked control of syllable sequence and phonology in birdsong. J. Neurosci..

[11] Kanwal JS, Matsumura S, Ohlemiller K, Suga N (1994). Analysis of acoustic elements and syntax in communication sounds emitted by mustached bats. J. Acoust. Soc. Am..

[12] Gadziola MA, Grimsley JM, Faure PA, Wenstrup JJ (2012). Social vocalizations of big brown bats vary with behavioral context. PLoS ONE.

[13] Prat Y, Taub M, Yovel Y (2016). Everyday bat vocalizations contain information about emitter, addressee, context, and behavior. Sci. Rep..

[14] Bohn KM, Schmidt-French B, Ma ST, Pollak GD (2008). Syllable acoustics, temporal patterns, and call composition vary with behavioral context in Mexican free-tailed bats. J. Acoust. Soc. Am..

[15] Wright GS, Chiu C, Xian W, Wilkinson GS, Moss CF (2013). Social calls of flying big brown bats (*Eptesicus fuscus*). Front. Physiol..

[16] Medvedev AV, Kanwal JS (2008). Communication call-evoked gamma-band activity in the auditory cortex of awake bats is modified by complex acoustic features. Brain Res..

[17] Washington SD, Kanwal JS (2008). DSCF neurons within the primary auditory cortex of the mustached bat process frequency modulations present within social calls. J. Neurophysiol..

[18] Ohlemiller KK, Kanwal JS, Suga N (1996). Facilitative responses to species-specific calls in cortical FM-FM neurons of the mustached bat. Neuroreport.

[19] Giraud AL, Poeppel D (2012). Cortical oscillations and speech processing: emerging computational principles and operations. Nat. Neurosci..

[20] Teng X, Tian X, Rowland J, Poeppel D (2017). Concurrent temporal channels for auditory processing: Oscillatory neural entrainment reveals segregation of function at different scales. PLoS Biol..

[21] Gross J (2013). Speech rhythms and multiplexed oscillatory sensory coding in the human brain. PLoS Biol..

[22] Hechavarria JC (2013). Blurry topography for precise target-distance computations in the auditory cortex of echolocating bats. Nat. Commun..

[23] Martin LM, Garcia-Rosales F, Beetz MJ, Hechavarria JC (2017). Processing of temporally patterned sounds in the auditory cortex of Seba's short-tailed bat, *Carollia perspicillata*. Eur. J. Neurosci..

[24] Esser KH, Eiermann A (1999). Tonotopic organization and parcellation of auditory cortex in the FM-bat *Carollia perspicillata*. Eur. J. Neurosci..

[25] Wehr M, Zador AM (2005). Synaptic mechanisms of forward suppression in rat auditory cortex. Neuron.

[26] Joris PX, Schreiner CE, Rees A (2004). Neural processing of amplitude-modulated sounds. Physiol. Rev..

[27] Buzsaki G, Anastassiou CA, Koch C (2012). The origin of extracellular fields and currents—EEG, ECoG, LFP and spikes. Nat. Rev. Neurosci..

[28] Fries P (2009). Neuronal gamma-band synchronization as a fundamental process in cortical computation. Annu. Rev. Neurosci..

[29] Rutishauser U, Ross IB, Mamelak AN, Schuman EM (2010). Human memory strength is predicted by theta-frequency phase-locking of single neurons. Nature.

[30] Magri C, Schridde U, Murayama Y, Panzeri S, Logothetis NK (2012). The amplitude and timing of the BOLD signal reflects the relationship between local field potential power at different frequencies. J. Neurosci..

[31] Ray S, Hsiao SS, Crone NE, Franaszczuk PJ, Niebur E (2008). Effect of stimulus intensity on the spike-local field potential relationship in the secondary somatosensory cortex. J. Neurosci..

[32] Chalk M (2010). Attention reduces stimulus-driven gamma frequency oscillations and spike field coherence in V1. Neuron.

[33] Martin KA, Schroder S (2016). Phase locking of multiple single neurons to the local field potential in Cat V1. J. Neurosci..

[34] Fries P, Reynolds JH, Rorie AE, Desimone R (2001). Modulation of oscillatory neuronal synchronization by selective visual attention. Science.

[35] Kayser C, Logothetis NK, Panzeri S (2010). Millisecond encoding precision of auditory cortex neurons. Proc. Natl Acad. Sci. USA.

[36] García-Rosales Francisco, Martin Lisa M., Beetz M. Jerome, Cabral-Calderin Yuranny, Kössl Manfred, Hechavarria Julio C. (2018). Low-Frequency Spike-Field Coherence Is a Fingerprint of Periodicity Coding in the Auditory Cortex. iScience.

[37] Kayser C, Montemurro MA, Logothetis NK, Panzeri S (2009). Spike-phase coding boosts and stabilizes information carried by spatial and temporal spike patterns. Neuron.

[38] Montemurro MA, Rasch MJ, Murayama Y, Logothetis NK, Panzeri S (2008). Phase-of-firing coding of natural visual stimuli in primary visual cortex. Curr. Biol..

[39] Beetz MJ, Hechavarria JC, Kossl M (2016). Temporal tuning in the bat auditory cortex is sharper when studied with natural echolocation sequences. Sci. Rep..

[40] Beetz M. Jerome, Kordes Sebastian, García-Rosales Francisco, Kössl Manfred, Hechavarría Julio C. (2017). Processing of Natural Echolocation Sequences in the Inferior Colliculus of Seba’s Fruit Eating Bat, Carollia perspicillata. eneuro.

[41] Syka J, Suta D, Popelar J (2005). Responses to species-specific vocalizations in the auditory cortex of awake and anesthetized guinea pigs. Hear. Res..

[42] Vicario DS, Yohay KH (1993). Song-selective auditory input to a forebrain vocal control nucleus in the zebra finch. J. Neurobiol..

[43] Schreiner CE, Urbas JV (1988). Representation of amplitude modulation in the auditory cortex of the cat. II. Comparison between cortical fields. Hear. Res..

[44] Creutzfeldt O, Hellweg FC, Schreiner C (1980). Thalamocortical transformation of responses to complex auditory stimuli. Exp. Brain Res..

[45] Elhilali M, Fritz JB, Klein DJ, Simon JZ, Shamma SA (2004). Dynamics of precise spike timing in primary auditory cortex. J. Neurosci..

[46] Grimsley JM, Shanbhag SJ, Palmer AR, Wallace MN (2012). Processing of communication calls in Guinea pig auditory cortex. PLoS ONE.

[47] Wallace MN, Shackleton TM, Anderson LA, Palmer AR (2005). Representation of the purr call in the guinea pig primary auditory cortex. Hear. Res..

[48] Hyafil A, Fontolan L, Kabdebon C, Gutkin B, Giraud AL (2015). Speech encoding by coupled cortical theta and gamma oscillations. eLife.

[49] García-Rosales Francisco, Martin Lisa M., Beetz M. Jerome, Cabral-Calderin Yuranny, Kössl Manfred, Hechavarria Julio C. (2018). Low-Frequency Spike-Field Coherence Is a Fingerprint of Periodicity Coding in the Auditory Cortex. iScience.

[50] Lakatos P (2005). An oscillatory hierarchy controlling neuronal excitability and stimulus processing in the auditory cortex. J. Neurophysiol..

[51] Luo H, Liu Z, Poeppel D (2010). Auditory cortex tracks both auditory and visual stimulus dynamics using low-frequency neuronal phase modulation. PLoS Biol..

[52] Panzeri S, Brunel N, Logothetis NK, Kayser C (2010). Sensory neural codes using multiplexed temporal scales. Trends Neurosci..

[53] Luo H, Poeppel D (2007). Phase patterns of neuronal responses reliably discriminate speech in human auditory cortex. Neuron.

[54] Arnal LH, Giraud AL (2012). Cortical oscillations and sensory predictions. Trends Cogn. Sci..

[55] Farahani ED, Goossens T, Wouters J, van Wieringen A (2017). Spatiotemporal reconstruction of auditory steady-state responses to acoustic amplitude modulations: Potential sources beyond the auditory pathway. Neuroimage.

[56] Krishna BS, Semple MN (2000). Auditory temporal processing: responses to sinusoidally amplitude-modulated tones in the inferior colliculus. J. Neurophysiol..

[57] Bartlett EL, Wang X (2007). Neural representations of temporally modulated signals in the auditory thalamus of awake primates. J. Neurophysiol..

[58] Gao L, Kostlan K, Wang Y, Wang X (2016). Distinct. Neuron.

[59] Suzuki TN, Wheatcroft D, Griesser M (2016). Experimental evidence for compositional syntax in bird calls. Nat. Commun..

[60] Belitski A, Panzeri S, Magri C, Logothetis NK, Kayser C (2010). Sensory information in local field potentials and spikes from visual and auditory cortices: time scales and frequency bands. J. Comput. Neurosci..

[61] Harris KD, Henze DA, Csicsvari J, Hirase H, Buzsaki G (2000). Accuracy of tetrode spike separation as determined by simultaneous intracellular and extracellular measurements. J. Neurophysiol..

[62] Lewicki MS (1998). A review of methods for spike sorting: the detection and classification of neural action potentials. Network.

[63] Ray S (2015). Challenges in the quantification and interpretation of spike-LFP relationships. Curr. Opin. Neurobiol..

[64] Desbordes G (2008). Timing precision in population coding of natural scenes in the early visual system. PLoS Biol..

[65] Goldberg JM, Brown PB (1969). Response of binaural neurons of dog superior olivary complex to dichotic tonal stimuli: some physiological mechanisms of sound localization. J. Neurophysiol..

[66] Berens P (2009). CircStat: a MATLAB toolbox for circular statistics. J. Stat. Softw..

[67] Grasse DW, Moxon KA (2010). Correcting the bias of spike field coherence estimators due to a finite number of spikes. J. Neurophysiol..

[68] Percival, D. B. & Walden, A. T. *Spectral Analysis for Physical Applications* (Cambridge University Press, Cambridge, 1993).

[69] Bokil H, Andrews P, Kulkarni JE, Mehta S, Mitra PP (2010). Chronux: a platform for analyzing neural signals. J. Neurosci. Methods.

[70] Shannon CE (2001). A mathematical theory of communication. ACM SIGMOBILE Mob. Comput. Commun. Rev..

[71] Montemurro MA, Senatore R, Panzeri S (2007). Tight data-robust bounds to mutual information combining shuffling and model selection techniques. Neural Comput..

[72] Magri C, Whittingstall K, Singh V, Logothetis NK, Panzeri S (2009). A toolbox for the fast information analysis of multiple-site LFP, EEG and spike train recordings. BMC Neurosci..

[73] Borst A, Theunissen FE (1999). Information theory and neural coding. Nat. Neurosci..

[74] Strong, S. P., de Ruyter van Steveninck, R. R., Bialek, W. & Koberle, R. On the application of information theory to neural spike trains. In: *Pacific Symposium on Biocomputing ’98*, RB, Dunker AK, Hunter L, and Klein TE. Maui, Eds., (HI: Singapore World Scientific, 1998a), 621–632. 9697217

[75] Panzeri S, Senatore R, Montemurro MA, Petersen RS (2007). Correcting for the sampling bias problem in spike train information measures. J. Neurophysiol..

[76] Benjamini Y, Hochberg Y (1995). Controlling the false discovery rate—a practical and powerful approach to multiple testing. J. R. Stat. Soc. B.

