# Supplementary material for: Neuronal coding of multiscale temporal features in communication sequences within the bat auditory cortex
Source: Commun Biol. 2018 Nov 20;1:200. doi: 10.1038/s42003-018-0205-5 (PMC6244232; doi:10.1038/s42003-018-0205-5)
Supplement: Supplementary file 1 — Supplementary Materials [file 42003_2018_205_MOESM1_ESM.pdf]

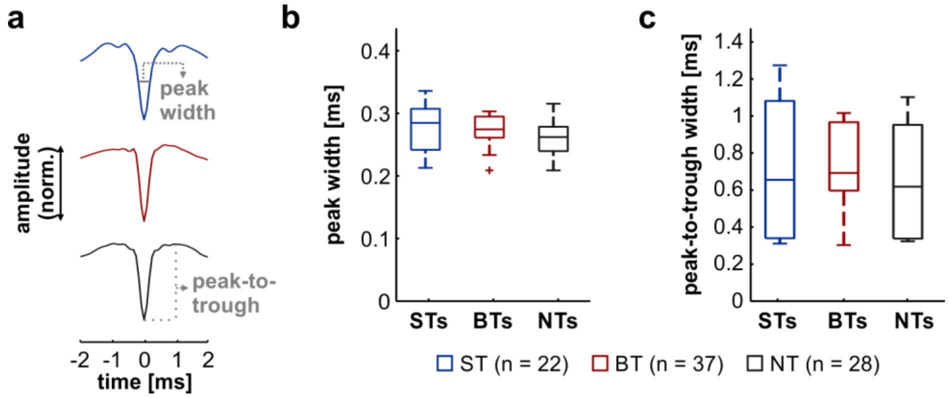

Supplementary Figure 1. Spike width does not significantly differ across neuronal groups. (**a**) Average spike shape of the exemplary ST (blue; top), BT (red; middle) and NT (black; bottom) units shown in **Fig. 2**. A total of 7992 spikes were considered for the ST unit, 7763 for the BT unit, and 2339 for the NT unit. (**b**) Comparison of the peak-width (note the representation in panel **a**) of ST, BT and NT units. There were no significant differences across groups (FDR-corrected Wilcoxon rank-sum tests, $p_{corr} > 0.11$). (**c**) Comparison of peak-to-trough width (note the representation in panel **a**) across neuronal groups. There were no significant differences ($p_{corr} > 0.43$) across groups

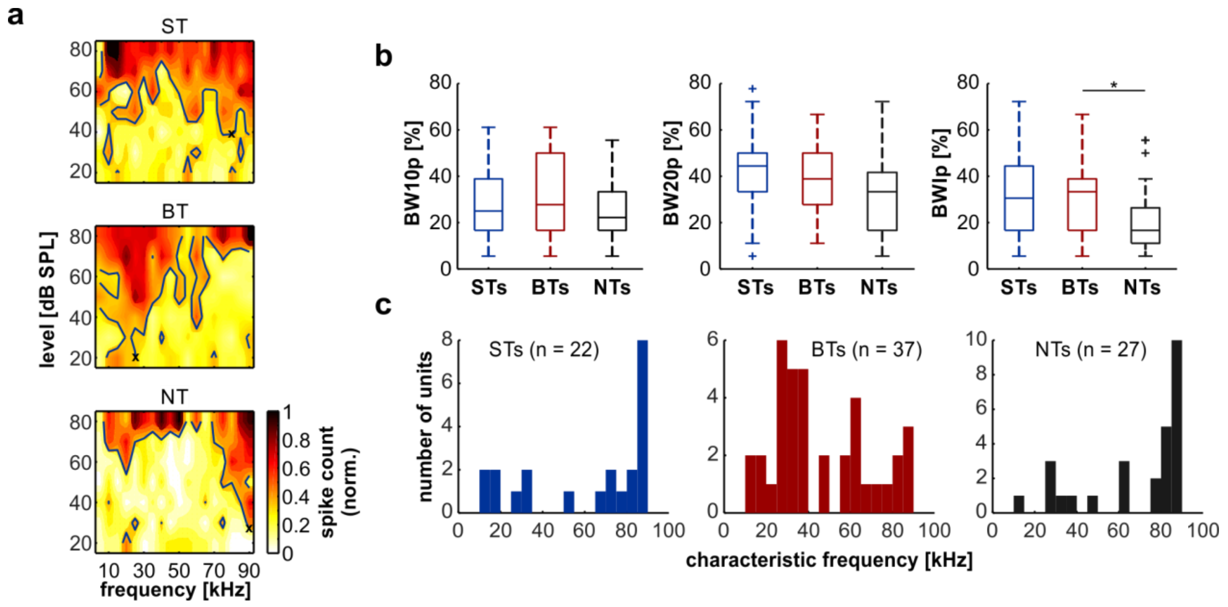

Supplementary Figure 2. Frequency-level receptive field properties across neuronal groups. (**a**) Frequency level receptive fields (RF) of an ST, a BT and a NT unit (same exemplary units shown in **Fig. 2**). A black cross in the heatmaps represents the characteristic frequency (CF; i.e. the frequency at which the unit was responsive with the lowest sound pressure level; note that the sound level at which the cross is marked is also the unit's minimum threshold). Contour lines indicate a response threshold of 33.3% of the maximum spiking response in the RF. (**b**) Quantification of the three metrics used to describe the broadness of the RFs, for each neuronal type (i.e. STs, n = 22; BTs, n = 37; and NTs, n = 27; note that one of the NT units could not be used for analyses because its frequency tuning file was corrupted). The left panel depicts comparisons of the proportion of frequencies in the RF that elicited responses above 33.3% of the maximum spiking (indicated in percent), considering responses to tones with a level 10 dB above the minimum threshold (BW10p) of the considered unit. The middle panel shows a similar metric but calculated considering spiking responses to sounds with a level 20 dB above the minimum threshold of the unit under consideration (BW20p). The right column quantifies differences in the overall activity in the RF across frequencies (i.e. integrating across all tested levels). In this case, the metric (BWIp) measures the percentage of tested frequencies for which the units spiked above 75% of the maximum response. Note that neuronal groups did not differ significantly in terms of their BW10p or BW20p (FDR-corrected Wilcoxon rank-sum tests; $p_{corr} > 0.21$). However, BT units had a significantly broader RF, in terms of BWIp, than ST units, but there were no significant differences between ST and BT units. Corrected p-values for the statistical comparisons are as follows: STs vs. BTs,

p_{corr} = 0.94; STs. vs. NTs, p_{corr} = 0.064; BTs vs. NTs, p_{corr} = 0.038). (**c**) Distribution of CFs for the three neuronal groups. In our data, 62.1 % (23/37) of BT units had CFs < 50 kHz (i.e. were tuned to low frequency), whereas only 31.8 % (7/22) of the ST units, and 25.9 % (7/27) of the NT units had were tuned to low frequencies. There were significant differences between the CF distribution of BTs and STs, and of BTs and NTs, but not between the CF distribution of ST and NT units (FDR-corrected 2 sample Kolmogorov-Smirnov tests; STs *vs* BTs, p_{corr} = 0.0154; STs *vs* NTs, p_{corr} = 0.9448; BTs *vs* NTs, p_{corr} = 0.0039). (*, $p_{corr} < 0.05$).

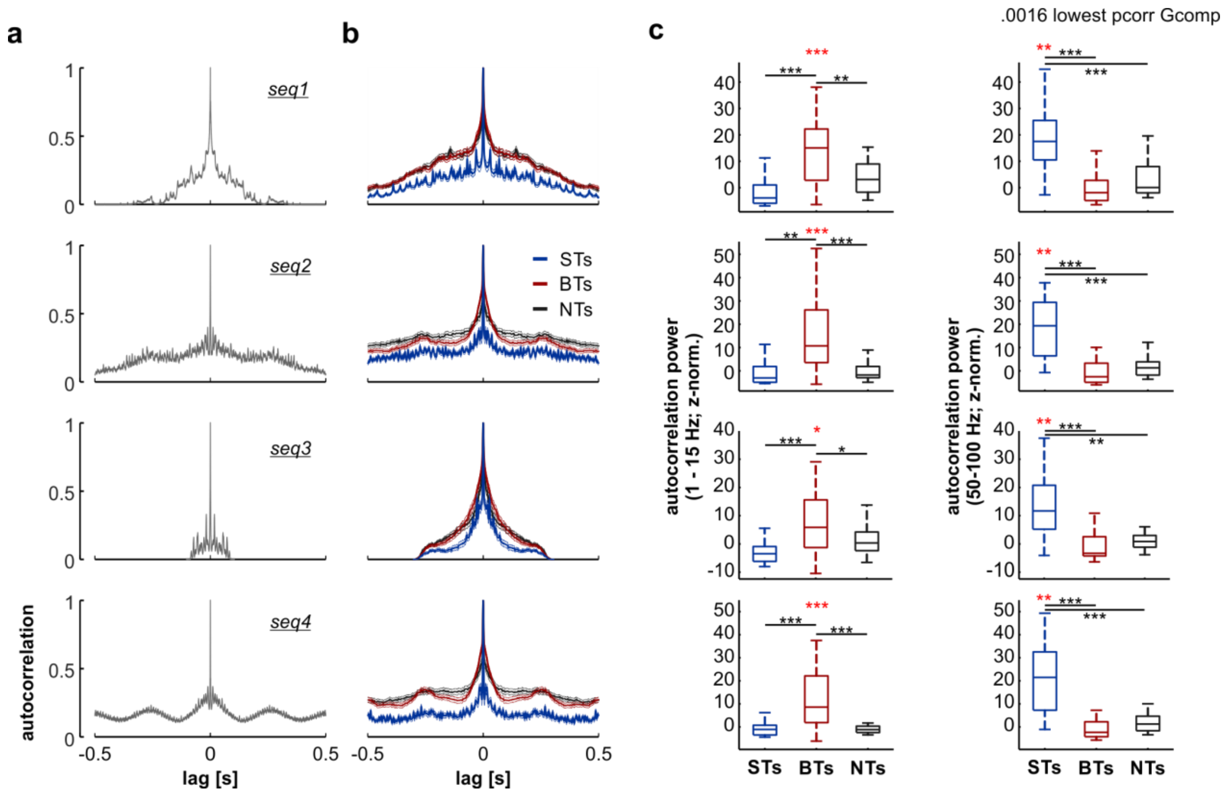

Supplementary Figure 3. Response periodicities are specific for syllable- and bout-tracking units. (**a**) Autocorrelation of the temporal envelopes of each natural sequence used as stimulus. Note the marked slow periodicity of seq4 (~250 ms cycle, equivalent to 4 Hz), which matches the bout periodicity of the call (8 bouts in ~2 seconds, also close to 4 Hz). (**b**) Autocorrelation of the spike probability density function (1 ms precision) for the populations of ST (blue), BT (red) and NT (black) units. Responses from BT units to seq4 show slow periodicities (250 ms cycle in the lags of the autocorrelation, or 4 Hz), whereas the autocorrelation of responses from ST units have faster dynamics. The same is seen (albeit less evidently) in response to other sequences. (**c**) Comparison of periodicities (see Methods) present in the autocorrelations obtained from the spiking activity. As a measure of periodicity, the z-normalized (to a surrogate distribution) low- or high-frequency power of the autocorrelation was used. The left column shows analyses for slow periodicities (1-15 Hz), while the right column shows results from analyses of fast periodicities (50-100 Hz) in the response, across calls. Bout-tracking units had a significant presence of low periodicities (FDR-corrected tailed Wilcoxon signed-rank tests; $p_{corr} <= 0.02$**;** red stars on top of boxplots indicate statistical significance; see Supplementary Methods for details) in their spiking responses, although that was not the case for ST or NT units ($p_{corr} > 0.24$). Furthermore, the z-normalized low frequency power of the

autocorrelation was higher in BT units than in ST or NT units, independently of the call under consideration (FDR-corrected Wilcoxon rank-sum tests, $p_{corr} <= 0.025$). On the other hand, ST units had a significance presence of high frequency periodicities in their response ($p_{corr} < 7.7x10^{-4}$; red stars on top of boxplots indicates statistical significance, see Methods for details), which was not true for either BT or NT units ($p_{corr} > 0.65$). Moreover, the fast temporal structure of the autocorrelation of ST units was significantly stronger than BT or NT units (FDR-corrected Wilcoxon rank-sum tests, $p_{corr} <= 1.4x10^{-3}$). (*, $p_{corr} < 0.05$; **, $p_{corr} < 0.01$; ***, $p_{corr} < 0.001$).

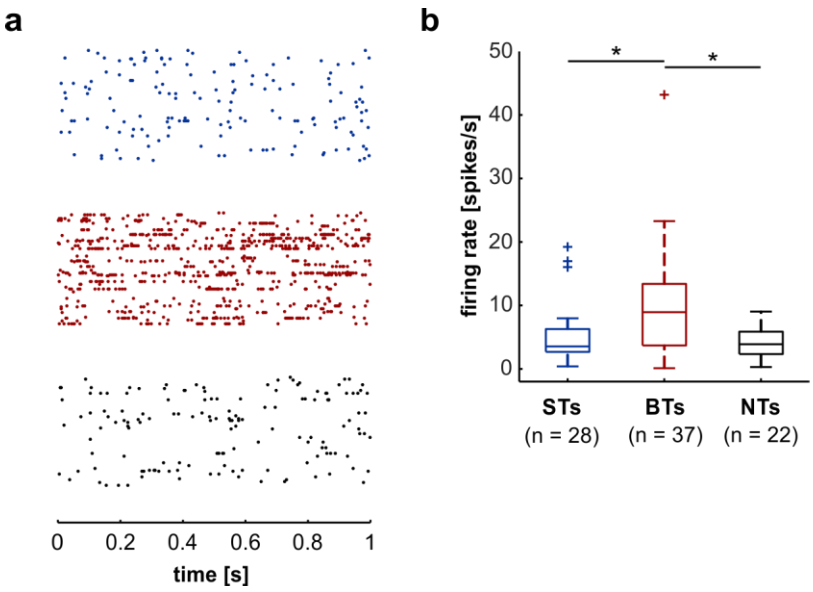

Supplementary Figure 4. Spontaneous spiking activity in recorded units. (**a**) Spontaneous activity (i.e. neuronal activity without auditory stimulation) was recorded for every unit. From top to bottom, the spontaneous spiking in the three example units depicted in **Fig. 2** is shown (top, ST unit; middle, BT unit; bottom, NT unit). The spikes were taken from consecutive, non-overlapping 1 s long epochs (over a total of 50 s) and were arranged according to the specific epoch in which they were detected from bottom to top. (**b**) Comparison of firing rate during spontaneous activity for the three neuronal groups. Bout-tracking units significantly differed from ST and NT units (FDR-corrected Wilcoxon rank-sum test, $p_{corr} <= 0.034$), whereas there were no significant differences between the groups of ST and NT units ($p_{corr} > 0.83$). (*, $p_{corr} < 0.05$).

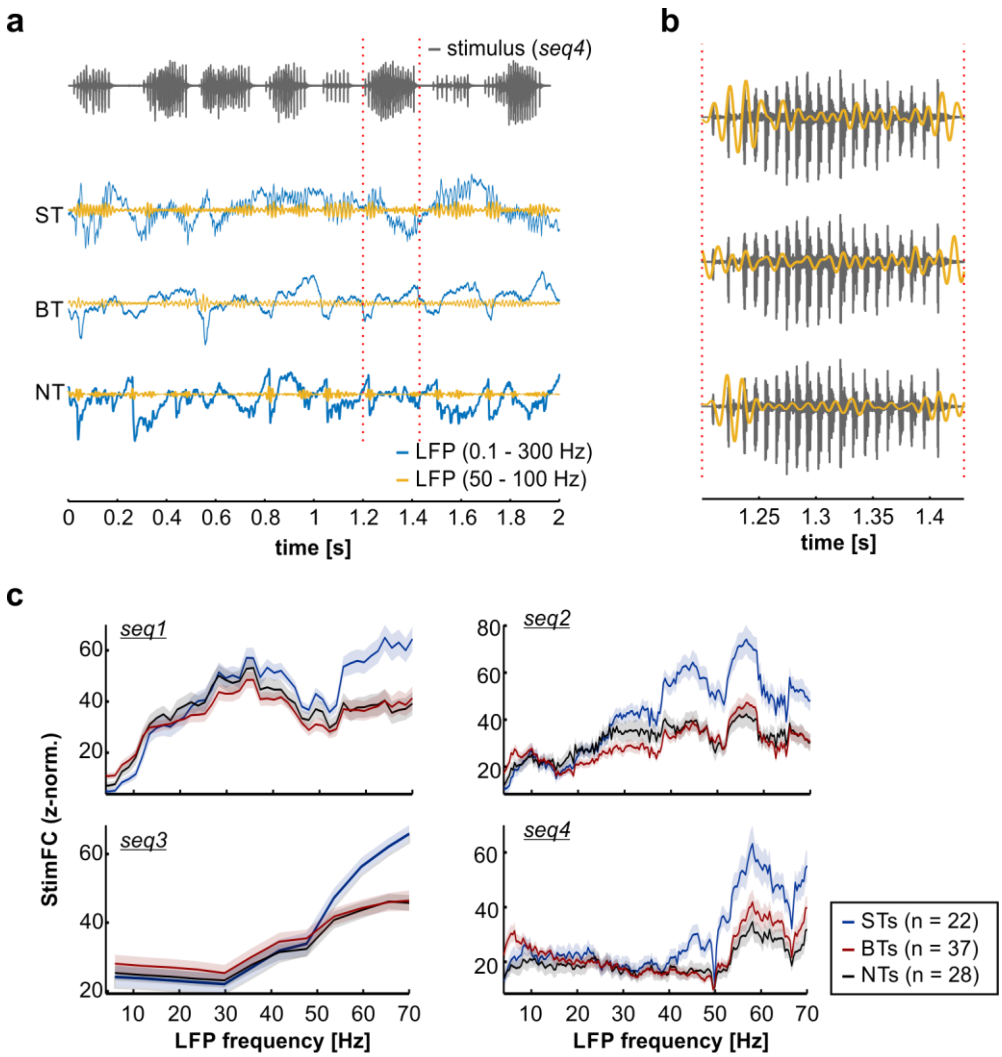

Supplementary Figure 5. High frequency LFPs synchronize to the calls. (**a**) Stimulus (gray) and average de-spiked LFP traces (over 50 trials) of the ST, BT and NT units shown in **Fig. 2**. Blue curves represent the broadband LFP (0.1 – 300 Hz), while yellow curves represent high frequency (50 – 100 Hz) oscillations. Data is shown in response to the same sequence depicted at the top of the panel (seq4). (**b**) Zoom-in of stimulus (gray) and high frequency LFPs (yellow) traces, between the time points demarcated in panel **a** by vertical red dashed lines (corresponding to bout 6 of the sequence). Note that fast LFPs align to the syllabic structure of the bout (Top: LFP from the ST unit; Middle: LFP from the BT unit; Bottom: LFP from the NT unit). (**c**) z-normalized stimulus-field coherence (StimFC) of the population of ST, BT and NT units (blue, red and black, respectively; shown as mean +- SEM), for all distress sequences tested in the study. Fast oscillations (> 50 Hz) were highly synchronized, indicating that LFPs were entrained by the rhythmicity of the syllabic rate in the sequences. The former was true independently of the units' classification, although

oscillations related to ST units typically had stronger values of coherence in high frequencies.

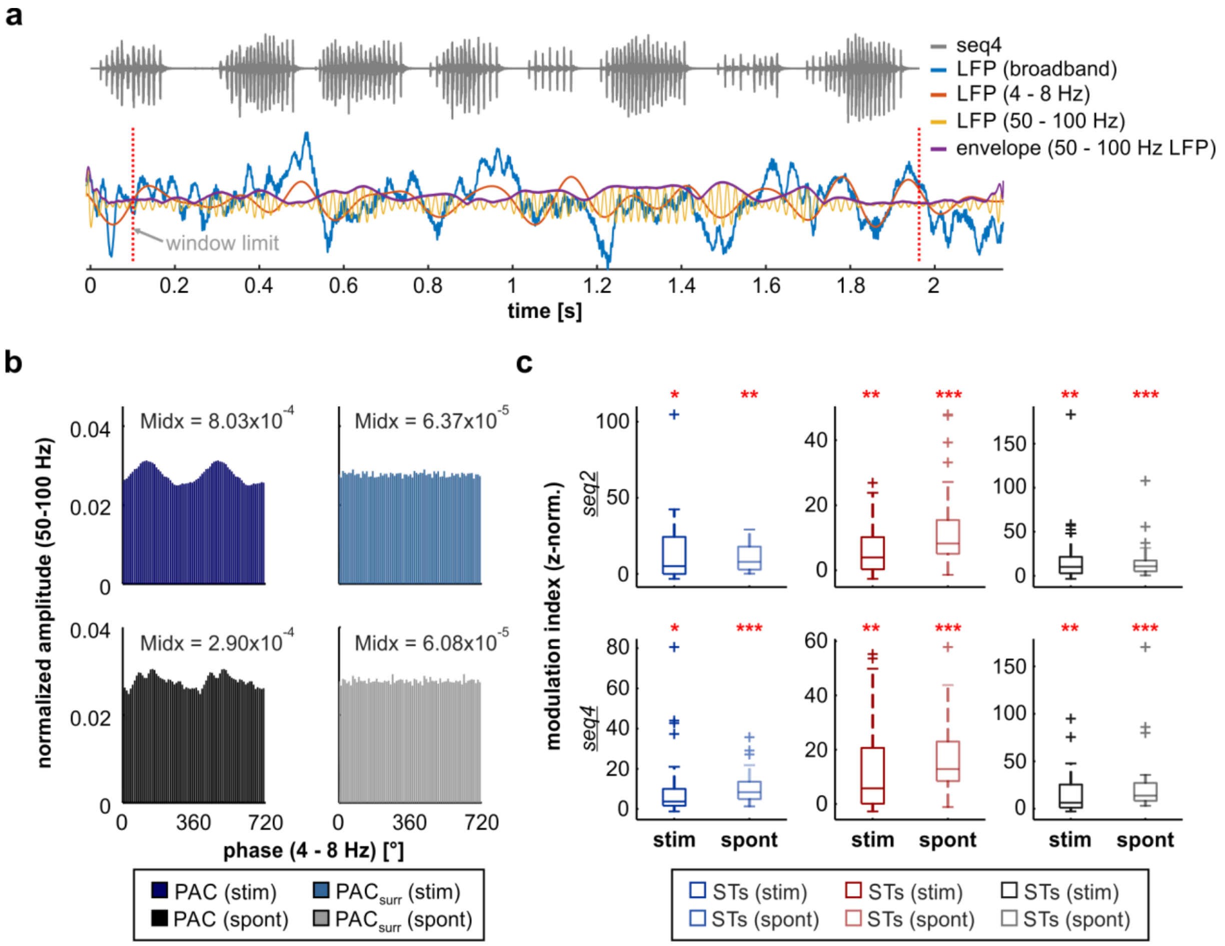

Supplementary Figure 6. Phase amplitude coupling (PAC) in ST, BT and NT units. (**a**) Stimulus (seq4, gray trace) and single trial LFP trace (broadband LFP, blue; 4 – 8 Hz LFP, orange; 50 – 100 Hz LFP, yellow; note the amplitude envelope of the 50 – 100 Hz LFP, depicted in purple), associated to the same BT unit shown in **Fig. 2c**, in response to seq4. The amplitude envelope of the 50 – 100 Hz (fast) LFP trace, and the phase of the 4 – 8 Hz (theta) wave were used for the PAC analyses. (**b**) Amplitude distribution of the fast LFP relative to the theta phase (two cycles are shown for illustrative purposes). The top row shows the amplitude across phase obtained from LFPs recorded in response to seq4, across trials, for the same unit shown in **A**. The observed distribution from the data is depicted on the left, whereas a surrogate amplitude distribution (from one repetition) is shown on the right (see Supplementary Methods). The bottom row follows the same conventions described above, but the amplitude distribution across phase bins is shown for the unit's spontaneous activity. The modulation indexes (Midxs) for each case are indicated in the figure. Note that, to allow for further PAC analyses and quantifications, Midxs from the observed data were z-normalized to a surrogate distribution (m = 500 repetitions), in which the relationship between the 50 – 100 Hz LFP amplitude was consistently disassociated

from the 4 – 8 Hz LFP phase (see Methods). (**c**) z-normalized Midxs across neuronal groups (STs, blue, n = 22; BTs, red, n = 37; and NTs, black, n = 22), in response to the two longest sequences used as stimuli (i.e. seq2 and seq4, strong line colors), and during spontaneous activity (light line colors). There was significant PAC between high frequency LFPs and theta band oscillations, during spontaneous activity and also while the animal listened to the calls, independently of the unit type considered (red stars on top of each boxplot indicate z-values significantly higher than 2; FDR-corrected Wilcoxon signed-rank tests; $p_{corr} <= 0.03$; *, $p_{corr} < 0.05$; **, $p_{corr} < 0.01$; ***, $p_{corr} < 0.001$). There were no significant differences between z-normalized Midxs during spontaneous activity and during sound stimulation (FDR-corrected Wilcoxon signed-rank tests; $p_{corr} > 0.08$), nor did we observe significant differences between neuronal groups (FDR-corrected Wilcoxon rank-sum tests; $p_{corr} > 0.27$), suggesting that in our data, the PAC between theta and fast LFPs was not a reliable marker to distinguish the temporal patterns observed in ST, BT or NT units.

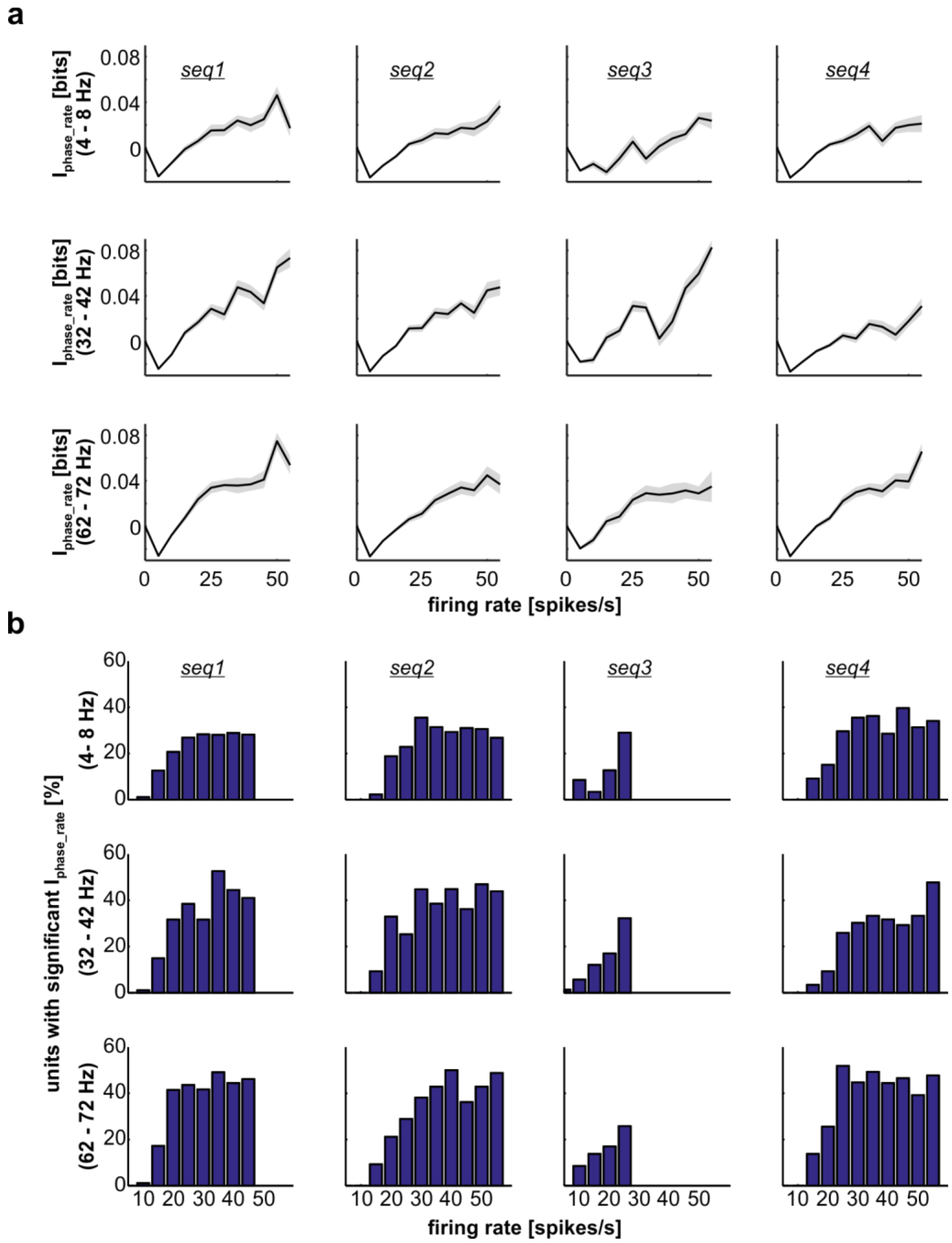

Supplementary Figure 7. Phase of low and high-frequency LFPs genuinely contribute to the information content of spiking rate. (**a**) The information in a fase-of-fire code (I_{rate_phase}) was quantified using sub-stimulus windows in which the firing rate of the unit was the same (see Supplementary Methods). Thus, the information provided by the unit's spiking rate (I_{rate}) was nullified, and the information provided by the phase of the LFP in the I_{rate_phase} code can be thought as novel relative to the firing rate. For the three LFP frequency bands tested (4 –8 Hz, 32 – 42 Hz, 62 – 72 Hz), and in response to the four natural sequences, we observed positive values of I_{rate_phase}, which typically increased together with the spike rate under consideration. The former indicates that phase of LFPs provide genuine additional information to the spiking,

and that this is true as well for high frequency (here, 62 – 72 Hz) oscillations. Data is shown as mean (solid line) +- SEM (shaded area; n = 87) (**b**) Percentage of the number of units for which I_{rate_phase} was significantly higher than 0, and I_{rate} was not (each compared to a surrogate distribution), as a function of fixed firing rate. Values are shown for the same three frequency bands of panel **a**, and in response to all natural calls used as stimuli. Note that firing rates >= 30 spikes/s are not shown for *seq3*, because not a sufficiently large proportion of the units (> 33 %, 29/87) could be considered for analyses.

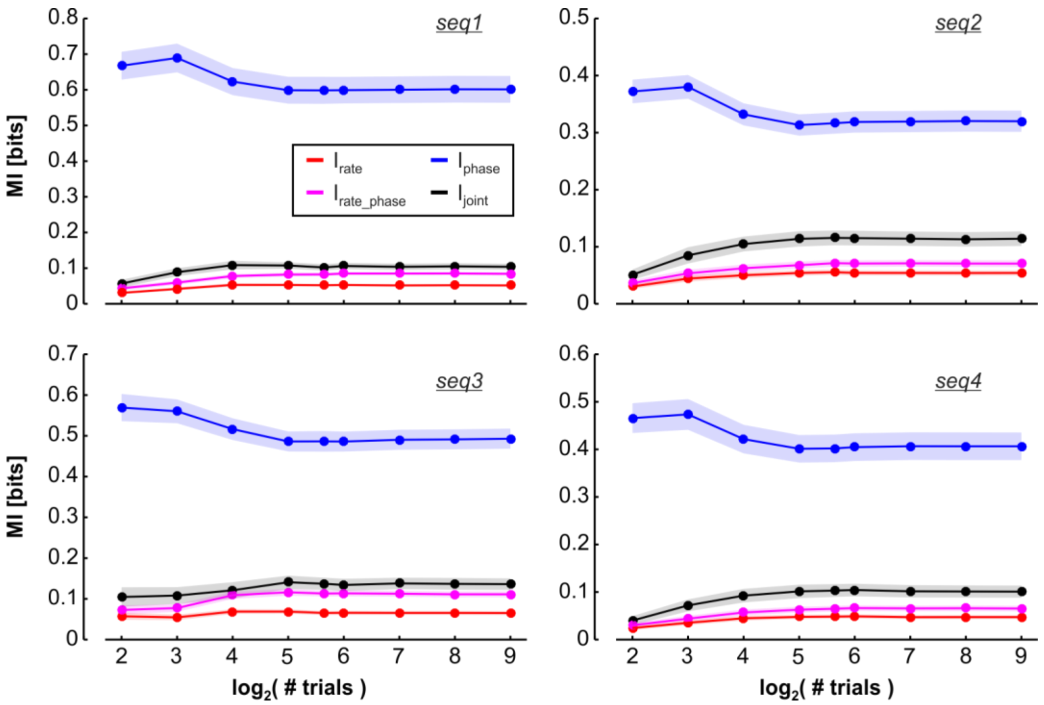

Supplementary Figure 8. Performance of bias correction methods for information theoretic analyses. In order to evaluate the performance of the bias correction methods used to calculate information values, we generated data with statistics close to the real experimental data and estimated the information in the neural codes considered (each colored trace in the figure; see legend). Each value was obtained using the same procedures as in the main analyses. When less than 32 trials were used, the bias was negative for codes involving neuronal spiking, and positive for I_{phase} (i.e. it was underestimated in the first case and overestimated in the second). Information estimates for I_{rate_phase} were affected the most (magenta trace) by the negative bias, which indicates that the information value in such code might have been slightly underestimated. Nevertheless, considering the number of trials used in our experiments (50), the bias is negligible and does not affect the conclusions of the study. Here, synthetic data was generated for all sequences tested, and the values for codes involving LFP phases are shown for the band of 62 – 72 Hz.

Supplementary Table 1. Basic temporal properties of the natural distress sequences used as stimulus. Basic temporal properties of the stimulus sequences used. Note that in the case of sequences 1 and 3, the whole call was considered as a bout for simplicity. The sound intensity was obtained, per sequence, considering each syllable individually.

seq	***# of bouts***	***# of syllables***	***Sequence length [s]***	***Avg. length (bouts) [ms]***	***Avg. length (syllables) [ms]***	***Avg. inter-syllable interval [ms]***	***Intensity (min., avg., max.) [dB SPL]***
1	1	9	0.51	510	69.2	141.4	64.5, 78.0, 81.6
2	7	53	1.46	140	4.8	26.4	64.6, 77.4, 84. 8
3	1	7	0.10	100	4.1	13.6	67.0, 72.6, 77.6
4	8	122	1.96	176.6	2.9	15.7	64.9, 77.5, 83.0

Supplementary Methods

Periodicity analysis of neuronal responses

Periodicities present in the responses from groups of ST, BT or NT units were assessed using the autocorrelation of the spiking PDFs', taking in to account that slow (or fast) periodicities would be marked by low (or high) frequency oscillations in the autocorrelogram. Formally, the above was addressed by calculating the power of the PDF's autocorrelation in low (0.1 – 15 Hz) and high (50 – 100 Hz) frequencies, on a unit per unit basis. Power values were z-normalized to a surrogate distribution in which the group-specific oscillatory patterns present in the autocorrelations were abolished, while still preserving the general properties of the autocorrelogram (e.g. the main peak at a lag of 0). In practice this was accomplished by randomly assigning labels (ST, BT or NT) to the units, and by calculating the average autocorrelogram of the resulting randomized groups. The power of such average autocorrelation in low and high frequencies was calculated and stored, and the procedure was repeated a total of 500 times. Ultimately, the distribution obtained from these values was used as a surrogate distribution of power in low and high frequencies, to which values observed in the data could be referenced. Note that labels were randomly assigned in a way that the size of the groups would be preserved (ST, 22 units; BT, 37 units; and NT, 28 units).

Responses of a certain neuronal group were considered to carry slow or fast periodicities if the z-normalized power in low or high frequencies of the set of units comprising the group were significantly higher than a z-score of 2 (FDR-corrected tailed Wilcoxon signed-rank tests; significance when $p_{corr} < 0.05$; red stars in **Supplementary Figure 3**). When comparing values across groups, FDR-corrected Wilcoxon rank-sum tests were performed (statistical significance for $p_{corr} < 0.05$).

Stimulus-field coherence

Coherence between cortical oscillations and the natural sequences was calculated using the stimulus-field coherence (StimFC) metric. Conceptually, the StimFC is similar to the SFC: a frequency-dependent, normalized synchronization index that quantifies how LFPs lock to the stimulus. In this case, LFP windows extend from the stimulus onset until its offset (note that this implies variations in LFP segment length across sequences). The average (across trials, n = 50) of the LFP windows (stimulus-triggered average, StimTA) retains oscillatory components whose phase is consistent throughout stimulus presentations, and therefore related to the stimulus, while non-synchronized components are "averaged-out". Similar to

the SFC calculations, the power of the StimTA is normalized to the average power of the individual LFP windows, which yields values between 0 and 1 indicating the strength of coherence in a certain frequency of the spectrum. LFP-stimulus synchronization was calculated on unit-per-unit basis, for every distress sequence analyzed. Power spectra were obtained using the multitaper[1] method available in Chronux[2], using 5 tapers and a TW product of 8.

The StimFC was z-normalized to a surrogate distribution in which phase consistencies across trials were destroyed as described below. For each trial (considering a certain unit and natural sequence), the LFP trace was split at a random time-point, and the resulting two segments were swapped. By doing this, surrogate StimTAs lost the synchronized oscillatory components present in the observed data. Based on the above, surrogate StimFC values, representing results with chance-level coherence, were calculated. The described procedure was repeated 500 times, in which surrogate values were computed with the same parameters used for the observed data. Finally, StimFC values were z-normalized, on a frequency bin basis, to the corresponding surrogate distribution (note that the above was done on a unit per unit basis, for all natural sequences tested).

Phase amplitude coupling

The phase amplitude coupling (PAC) analyses were performed as described in [3], on a unit per unit basis, using LFPs recorded in response to sequences 2 and 4 (with LFP windows extending from 100 ms after stimulus onset, until stimulus offset). Other sequences (1 and 3) were not used because their lengths did not allow for a robust estimation of PAC. In each case, the phase modulator was considered to be the theta-band (4 – 8 Hz) LFP, whereas the signal whose amplitude was being modulated was considered to be the fast (50 – 100 Hz) LFP. The instantaneous phase of theta-band oscillations, as well as the amplitude envelope of fast LFPs, were obtained from the Hilbert transform of the respective filtered LFP traces [3], and were paired in a composite time series $[\phi(t), A(t)]$. Phases were then binned into 36 equally sized bins (10° angular size), and for each bin j we accumulated the mean instantaneous energy of the fast LFP ($A(t)$), coupled with phases that fell within that particular bin. The amplitude of each bin was normalized to the sum across bins, which results in a distribution that holds the properties of a discrete probability density function. The former is referred to as “amplitude distribution” [3]. The modulation index (Midx) was then obtained by normalizing the observed amplitude distribution (P) with a uniform distribution (U), by means of the Kullback-Leibler distance. Thus, the Midx ranges from 0 to 1 representing,

respectively, absolute absence of modulation, or perfect modulation. Similar analyses were performed for LFPs recorded in spontaneous activity, using randomly chosen LFP windows of the same length as those used for estimating PAC during sound stimulation.

To test for the significance of the observed PAC, we computed a surrogate distribution of Midx values from data in which the relationship between the phase of theta-band LFPs and the amplitude of fast oscillations was reduced to chance. The above was accomplished by block-swapping instantaneous phase traces across trials at random time points 500 times. Such procedure yields surrogates in which there is minimal distortion of the phase-amplitude dynamics [4], and was performed on a unit per unit basis, for all sequences tested and the spontaneous activity. The Midx values of each unit were then z-normalized to the corresponding surrogate Midx distributions. We considered a neuronal subgroup (i.e. ST, BT or NT units) to have significant PAC, if the values of z-normalized Midx of its units were significantly higher than 2 z-scores (FDR-corrected Wilcoxon signed-rank test; significance after $p < 0.05$).

Information theoretic analyses

Stimulus characterization

In order to quantify how much information auditory cortical units provided about the natural distress sequences, we calculated how informative these units were in terms of their ability to distinguish specific parts of the acoustic stimulus [5-8]. To that end, the sequence used as stimulus was divided into a set of s_k (k = 1, 2, 3, …, M) consecutive and non-overlapping segments (time windows used for calculating main results were of length T = 4 ms), and each of this sub-stimulus s was treated as an independent member of the set S (i.e. the whole divided sequence). The former makes no assumptions about which features of the call elicited a response, and therefore is well suited to quantify the information content of the responses to the sequences. Note that it is assumed that all sub-stimuli s_k are equiprobable.

Information in neural codes

To quantify the information contained in the firing rate of individual units (I_{rate}), we determined the number of spikes occurring in response to each of the sub-stimuli (s_k) considered and based on that we then computed the information content. In this case, the response set was defined as the occurrence or not of a spike ($R = \{0, 1\}$), and $P(r)$ was then the probability of firing (or not) in each unit. $P(r)$ was estimated throughout the total set of 50

trials used for stimulation. We considered the time window for the sub-stimuli (T = 4 ms) to be sufficiently short as to assume that only one spike would occur inside of them in a given trial, and thus the information estimates provided in this manuscript were calculated with binarized responses (i.e. only the occurrence of a spike was noted, not the total amount of spikes in a certain window). Note that the former assumption can only underestimate the total value of I_{rate}, but we verified that there was no significant information loss due to the response binarization by comparing information estimates with and without the binarizing the response (Wilcoxon sign-rank tests, $p_{corr} > 0.09$).

In order to compute the information provided by the LFP phase (I_{phase}), we filtered the LFP in 11 different and non-overlapping frequency bands, ranging from 4 to 72 Hz, using a 4th order Butterworth filter. The bandwidth was of 4 Hz for lower bands (i.e. 4 – 8, 8 – 12… and 28 – 32 Hz), and of 10 Hz for higher bands (i.e. 32 – 42 up to 62 – 72 Hz). The instantaneous phase of LFP in each band was obtained from the Hilbert transform of the filtered signals. The phase values within each sub-stimulus were used to quantify I_{phase}. Because the phase is a continuous quantity, its values within a particular stimulus were binned into ϕ equally-sized bins. Main results were obtained using $\phi = 4$ bins because this number was found to be optimal (regarding the tradeoff between the information loss due to the discretization and the bias introduced by finer bin sizes) in previous studies [7,8], and because it allows to reliably quantify information in high frequency oscillations. In practice, each sub-stimulus was labeled with a symbol representing the corresponding bin of the mean phase across all instantaneous phases within the sub-stimulus. In other words, the response set took values of $R = \{1, 2, \ldots \phi\}$.

For the rate and phase information (I_{rate_phase}), a spike in a sub-stimulus was labeled with one of the ϕ possible symbols in which the average instantaneous phase (within the sub-stimulus) was binned. This way, spikes were associated to a certain phase bin of the LFP in each of the frequency bands analyzed, yielding a set of responses that can be represented as $R = \{0, 1, 2, \ldots, \phi\}$, where 0 implies the occurrence of no spike, whereas symbols 1 - ϕ indicate the occurrence of a spike, labeled with a certain phase bin.

If adding the phase of the LFP to the spike rate provides additional information not contained in the rate alone, and this information is indeed genuine (i.e. the phase of the LFP is not a function of the spiking rate), then the information in I_{rate_phase} must be still higher than 0, even when the I_{rate} is nullified. The latter was practically achieved by selecting sub-stimuli in which

the spike rate was the same (rendering this particular code as non-informative) and quantifying the information in I_{rate_phase}. As mentioned above, observing an $I_{rate\_phase} > 0$ would be a way to verify that the phase-of-firing at a particular frequency band of the LFP provides additional and genuine information not contained in the rate-of-firing alone [7,8]. The procedure of selecting stimulus epochs in which the units fired with the same rate was done for rates up to 55 spikes/s. Rates higher than 55 spikes/s were not considered because no more than 33% of the units (29 units) could be used for analyses.

We calculated the percentage of recorded units that significantly exhibited an increase in information when considering I_{rate_phase} in each fixed spiking rate. Our criterion was that a unit showed a significant increase in the rate-phase code if: i) the unit's I_{rate_phase} value considering a particular fixed spiking rate was significantly higher (z-score > 1.67, equivalent to a p-value of 0.05 of a tailed test) than a surrogate distribution; and ii) the unit's information content in I_{rate} was not significantly higher than the corresponding surrogate distribution. In other words, those criteria add up to considering a significant increase per unit if the information in I_{rate_phase} was higher than 0 *and* the information in I_{rate} was not.

We also quantified the information content in the response of two simultaneously recorded units to the presented natural vocalizations (I_{joint}). To that end, we used a spatial code which takes into account the identity of the unit that elicited (or not) a spike. The response in this case is described by four symbols, representing in binary all possible combinations of firing or not a spike considering each of the units (i.e. the response set is defined by $R = \{ (0, 0), (1, 0), (0, 1), (1, 1) \}$). Note that each pair in R is represented in the form $(r1, r2)$, where $r1$ and $r2$ each represent whether the first neuron or the second neuron in the pair fired or not a spike (1, a spike was elicited; 0 otherwise), respectively.

Supplementary References

1 Percival, D. B. & Walden, A. T. *Spectral analysis for physical applications*. (cambridge university press, 1993).

2 Bokil, H., Andrews, P., Kulkarni, J. E., Mehta, S. & Mitra, P. P. Chronux: a platform for analyzing neural signals. *Journal of neuroscience methods* **192**, 146-151 (2010).

3 Tort, A. B., Komorowski, R., Eichenbaum, H. & Kopell, N. Measuring phase-amplitude coupling between neuronal oscillations of different frequencies. *J Neurophysiol* **104**, 1195-1210, doi:10.1152/jn.00106.2010 (2010).

4 Aru, J. *et al.* Untangling cross-frequency coupling in neuroscience. *Curr Opin Neurobiol* **31**, 51-61, doi:10.1016/j.conb.2014.08.002 (2015).
5 de Ruyter van Steveninck, R. R., Lewen, G. D., Strong, S. P., Koberle, R. & Bialek, W. Reproducibility and variability in neural spike trains. *Science* **275**, 1805-1808 (1997).
6 Kayser, C., Logothetis, N. K. & Panzeri, S. Millisecond encoding precision of auditory cortex neurons. *Proc Natl Acad Sci U S A* **107**, 16976-16981, doi:10.1073/pnas.1012656107 (2010).
7 Kayser, C., Montemurro, M. A., Logothetis, N. K. & Panzeri, S. Spike-phase coding boosts and stabilizes information carried by spatial and temporal spike patterns. *Neuron* **61**, 597-608, doi:10.1016/j.neuron.2009.01.008 (2009).
8 Montemurro, M. A., Rasch, M. J., Murayama, Y., Logothetis, N. K. & Panzeri, S. Phase-of-firing coding of natural visual stimuli in primary visual cortex. *Curr Biol* **18**, 375-380, doi:10.1016/j.cub.2008.02.023 (2008).
